# Changes of the lipid membrane structures caused by chain-length-dependent doxorubicin embedment in PEGylated liposomes

**DOI:** 10.1107/S1600576725003577

**Published:** 2025-05-29

**Authors:** Jia-Jhen Kang, Zhih-Chen Huang, Li-Wen Tang, Chun-Jen Su, Hua-De Gao, Hsien-Ming Lee, U-Ser Jeng

**Affiliations:** a National Synchrotron Radiation Research Center Hsinchu 300092 Taiwan; b Institute of Chemistry Academia Sinica Taipei 115024 Taiwan; c Department of Chemical Engineering National Tsing Hua University Hsinchu 300044 Taiwan; d College of Semiconductor Research National Tsing Hua University Hsinchu 300044 Taiwan; European Molecular Biology Laboratory, Hamburg, Germany

**Keywords:** PEGylated liposomes, lipid chain lengths, bilayer structure, asymmetric flow field-flow fractionation, AF4, simultaneous small- and wide-angle X-ray scattering, SWAXS

## Abstract

Poly(ethyl­ene glycol)-grafted (PEGylated) liposomes receive increasingly more attention due to their practical applications in delivering vaccines, nutrients and drug molecules such as doxorubicin (DOX). PEGylated liposomes have been well documented for their capability in carrying DOX as rod-like crystallites enclosed inside the unilamellar vesicles. This study addresses the previously unresolved question of whether DOX intercalates into liposome bilayers by employing simultaneous small- and wide-angle X-ray scattering (SWAXS), complemented by an integrated asymmetric flow field-flow fractionation system coupled with multi-angle light scattering, dynamic light scattering and refractive index detection. The DOX-loaded PEGylated liposomes used are composed of phosphatidylcholine (*N*:0 PC) lipids, with different lipid chain lengths *N* = 18, 20 and 22, and a fixed molar ratio of lipid:cholesterol:DSPE-PEG2000 of 45:50:5. SWAXS analysis reveals that rod-like DOX nanocrystallites—approximately 70–95 nm in length and 14 nm in diameter—are encapsulated within the PEGylated liposomes across all three lipid types, with each exhibiting distinct membrane structural responses to DOX incorporation. Notably, 22:0 PC liposomes demonstrate significant DOX-induced disruption of lipid chain packing, accompanied by enhanced alignment of phosphate headgroups in the outer leaflet. Consistently, cryo-EM imaging reveals pronounced faceted membrane morphologies in DOX-loaded 22:0 PC liposomes. This faceting phenomenon is attributed to the accumulation of DOX within the excess hydro­phobic core regions created by the extended aliphatic chains beyond the cholesterol saturation limit. These DOX-enriched domains locally stiffen the membrane, promoting the formation of rigid, faceted structures.

## Abstract 2

Chain-length-dependent membrane changes in PEGylated liposomes induced by doxorubicin loading are revealed using small- and wide-angle X-ray scattering and cryo-EM.

## 1. Introduction

PEGylated liposomes are phospho­lipid-based vesicles that serve as functionalized nanocarriers (Ali Mohammadi *et al.*, 2016 ▸; Makwana *et al.*, 2021 ▸; Aloss & Hamar, 2023 ▸). In application, they show promising efficacy in the delivery of the anti-cancer drug doxorubicin (DOX), with several PEGylated liposome formulations already approved for clinical use. These liposomes are typically composed of a mixture of ‘helper lipids’ conjugated with polyethyl­ene glycol (PEG) to enhance solubility and structural stability, alongside cholesterol, which improves thermal stability and membrane fluidity (Geisler *et al.*, 2020 ▸; Nakhaei *et al.*, 2021 ▸; Shoji *et al.*, 1998 ▸). The amphiphilic nature of lipids leads to the formation of a bilayer membrane, which separates the inner hydro­philic core from the environment. To encapsulate DOX into the core region of PEGylated liposomes, the remote active loading method is the most widely used approach because of its high loading efficiency. It involves a transmembrane pH gradient, causing the penetration of DOX molecules through the membrane into the core part of the liposome (Haran *et al.*, 1993 ▸; Fritze *et al.*, 2006 ▸).

Earlier studies have established a pathway of DOX uptake into liposomes (Li *et al.*, 2018 ▸). It is found that DOX can form crystalline rod-like structures when DOX loading is performed in ammonium sulfate buffer (Lasic *et al.*, 1992 ▸; Schilt *et al.*, 2021 ▸), whereas round and curved DOX crystallites are formed in citrate buffer (Li *et al.*, 1998 ▸). Further investigations on the structural characterization of drug-free liposomes as well as DOX-loaded ones reveal a shape deformation of liposomes upon DOX loading (Xiao *et al.*, 2019 ▸; Nordström *et al.*, 2021 ▸). Also, the thicknesses of the membrane bilayer and the periphery PEG layers were precisely resolved (Schilt *et al.*, 2016 ▸; Li *et al.*, 2023 ▸). While the structure of the liposome bilayers and that of the DOX nanocrystallites embedded in liposomes have been extensively studied, the effects of DOX loading on the liposomal membrane structures remain not fully clarified. Considering the reported thickness of liposome membranes, typically between 3.5 and 5 nm (Su *et al.*, 2013 ▸; Su *et al.*, 2018 ▸; Schilt *et al.*, 2016 ▸), and the dimensions of DOX molecules, around 1.5 nm in length (Bilalis *et al.*, 2016 ▸), a critical question arises: how does DOX traverse the lipid bilayer to precipitate into crystallites within the liposome? This inquiry extends to whether DOX molecules intercalate within the membrane bilayers, potentially altering the membrane properties. Currently, the literature addressing these mechanisms remains sparse.

Small-angle X-ray scattering (SAXS) is a powerful non-invasive tool for characterizing liposomal structures, particularly at the nanoscale (Caselli *et al.*, 2024 ▸). SAXS is capable of revealing the electron density profile across a liposome bilayer in nano-resolution, which is critical for observing small changes of the bilayer structure and the structural impact of liposome composition modification. For SAXS data analysis of the bilayer structure, a widely accepted model is the multi-layered model, which generally consists of two high-electron-density layers corresponding to the phosphate headgroup regions, with a lower-electron-density region sandwiched in-between that corresponds to the hydro­phobic aliphatic chain region (Brzustowicz & Brunger, 2005 ▸; Varga *et al.*, 2010 ▸; Székely *et al.*, 2010 ▸). However, there are certain limitations when studying DOX-loaded liposomes. The overlapping scattering signals from DOX crystallites enclosed within the liposomes and the lipid bilayers themselves complicate the interpretation of the data. Separating these contributions remains a significant challenge. To address this, previous studies typically relied on modeling techniques that involve fitting SAXS data to a combined scattering profile from both the DOX crystallites and the lipid bilayer structures. This approach helps to approximate the contributions of each component but does not fully resolve them independently.

In order to gain a comprehensive understanding of how the DOX loading process affects the liposome’s membrane structure, in the present study, we systematically study the membrane structures of liposomes composed of phosphatidyl­choline lipids (*N*:0 PC, where *N* is the number of carbon atoms in the aliphatic chains), cholesterol and DSPE-PEG2000, without (pristine) and with DOX loaded. Here, DSPE-PEG2000 represents the helper lipid 1,2-distearoyl-*sn*-*glycero*-3-phospho­ethano­lamine (DSPE) conjugated with PEG with a molar mass of 2000 Da. These liposomes (with *N* = 18, 20 or 22) of a fixed molar ratio of lipid:cholesterol:DSPE-PEG2000 of 45:50:5 are characterized by simultaneous small- and wide-angle X-ray scattering (SWAXS), capable of resolving the bilayer structures including lipid chain packing features. In addition, integrative techniques of asymmetric flow field-flow fractionation (AF4) coupled to multi-angle light scattering (MALS), dynamics light scattering (DLS) and refractive index (RI) spectroscopies, as well as cryo-electron microscopy (cryo-EM), are utilized, for complementary structural information on the DOX-loaded liposomes. By employing this integrative approach, we correlate the structural changes of the liposome membrane with the lipid-chain-length-dependent DOX associations.

## 2. Materials and methods

### 2.1. Sample preparation

Liposomes were prepared by the thin-film hydration method followed by freeze–thaw and extrusion techniques (Lasic *et al.*, 1992 ▸; Schilt *et al.*, 2021 ▸). For the pristine liposomes (liposomes before DOX loading), prescribed formulations of phosphatidylcholine lipids (*N*:0 PC)/cholesterol/DSPE-PEG2000 with a molar ratio of 45:50:5 (*N* = 18, 20 or 22) were dissolved in chloro­form. Lipid films (8.75 µmol) were formed by rotary evaporation at 50 °C for 10 min and then left under vacuum overnight to remove residual solvent. The lipid films were hydrated in 1 ml of 250 m*M* ammonium sulfate, (NH_4_)_2_SO_4_, solution at specific temperatures (60 °C for the 18:0 PC formulation, 71 °C for the 20:0 PC formulation and 80 °C for the 22:0 PC formulation). The liposome suspensions were subjected to over ten freeze–thaw cycles in a liquid nitro­gen/60 °C water bath and subsequently extruded 21 times using a Mini Extruder (Avanti Polar Lipids) through track-etch 100 nm polycarbonate membranes at specific temperatures (60 °C for the 18:0 PC formulation, 71 °C for the 20:0 PC formulation and 80 °C for the 22:0 PC formulation) to obtain uniform-sized liposomes. The liposome solutions were then purified using Sepharose CL-4B equilibrated with 150 m*M* NaCl solution. For DOX liposomes, DOX was loaded into the pristine liposome solutions by mixing them at a drug-to-lipid ratio of 1:10 and incubating at 65 °C for 40 min. Finally, the DOX-encapsulated liposomes were purified and separated from non-encapsulated DOX using Sepharose CL-4B equilibrated with tricine buffer (50 m*M* tricine, 100 m*M* NaCl pH 7.5).

### 2.2. Cryo-electron microscopy

The cryo-EM imaging of the liposomes was conducted at the Cryo-TEM Core Facility, ICOB, Academia Sinica (Taipei, Taiwan). In brief, 200-mesh copper grids (HC200-Cu, PELCO) were glow-discharged for 15 s in an atmosphere of argon and oxygen (Ar, O_2_) on the carbon side. A 4 µl droplet of the liposome solution (with a final lipid concentration of 0.7 m*M*) was then applied to the grids. The grids were blotted at 100% humidity and 4 °C for 3–4 s before being plunge-frozen in liquid ethane, which was pre-cooled by liquid nitro­gen, using a Vitrobot (FEI, Hillsboro, OR, USA). Imaging was performed on an FEI Tecnai G2 F20 TWIN transmission electron microscope at 200 keV. Transmission electron microscopy was carried out in bright-field mode with an operating voltage of 200 kV. Images were captured with a defocus of approximately 1.8 µm under low-dose conditions (25–30 e^−^ Å^−2^) using a 4k× CCD camera (Gatan, Pleasanton, CA, USA) at a magnification of 50000×.

### 2.3. AF4-MALS-DLS-RI

The liposome hydro­dynamic size and particle mass characterizations were performed using an AF4 system coupled with a MALS detector and a differential RI detector, utilizing the Wyatt Eclipse DualTec instrumentation system, where DLS data were obtained simultaneously from one of the 18 detectors of the MALS machine (Shih *et al.*, 2022 ▸). The AF4 separation channel consisted of a trapezoidal spacer of 265 mm in length and 35 µm in height and a regenerated cellulose ultrafiltration membrane with a molecular weight cutoff of 10 kDa. Each measurement involved an injection sample volume of 10 µl of solution, eluted with phosphate-buffered saline (PBS). Detailed flow rate parameters are documented in Table S1 in the supporting information. The *ASTRA* program (Wyatt Technology) was employed for data analysis, with the refractive-index increment d*n*/d*c* = 0.146 ml g^−1^ separately measured for the cholesterol-incorporated, PEGylated liposomes without DOX, to convert the measured RI values to the concentrations of the liposomes (Hsu *et al.*, 2023 ▸); these were used in the Zimm model for particle mass calculation.

### 2.4. Small- and wide-angle X-ray scattering

SWAXS measurements were performed at the 13A Bio­SWAXS beamline of Taiwan Photon Source (TPS) at the National Synchrotron Radiation Research Center (Shih *et al.*, 2022 ▸). With an X-ray energy of 15 keV (wavelength λ = 0.8266 Å), SAXS and wide-angle X-ray scattering (WAXS) data were simultaneously collected by the Eiger X 9M and X 1M detectors, which were positioned in vacuum with sample-to-detector distances of 2500 and 300 mm, respectively. The scattering vector magnitude *q* = 4π sin θ/λ defined by the scattering angle 2θ and λ. SAXS and WAXS data were calibrated concomitantly using a mixed powder of silver behenate and lanthanum hexaboride (LaB_6_). The SAXS absolute intensity (in units of cm^−1^) was calibrated using water scattering intensity; the concomitantly measured WAXS data were aligned to the SAXS data for the absolute intensity and then normalized by the sample concentrations for quantitative comparison (Shih *et al.*, 2022 ▸). With an auto sample injection system, a constant flow of PBS buffer with a flow rate of 0.2 ml min^−1^ delivered the injected 150 µl of the sample solution through a quartz capillary (2 mm diameter with 10 µm wall thickness) for X-ray exposure. With the continuous flow mode, radiation damage of the liposomes was not observed from the data collected over the sample elution peak with exposure times of 2 s per data frame for more than 20 data frames. Buffer scattering was measured before and after the sample elution peak and used in buffer scattering subtraction. We note that the AF4 system was not coupled to the SWAXS measurements.

### 2.5. SAXS data analysis

**Figure 1:**
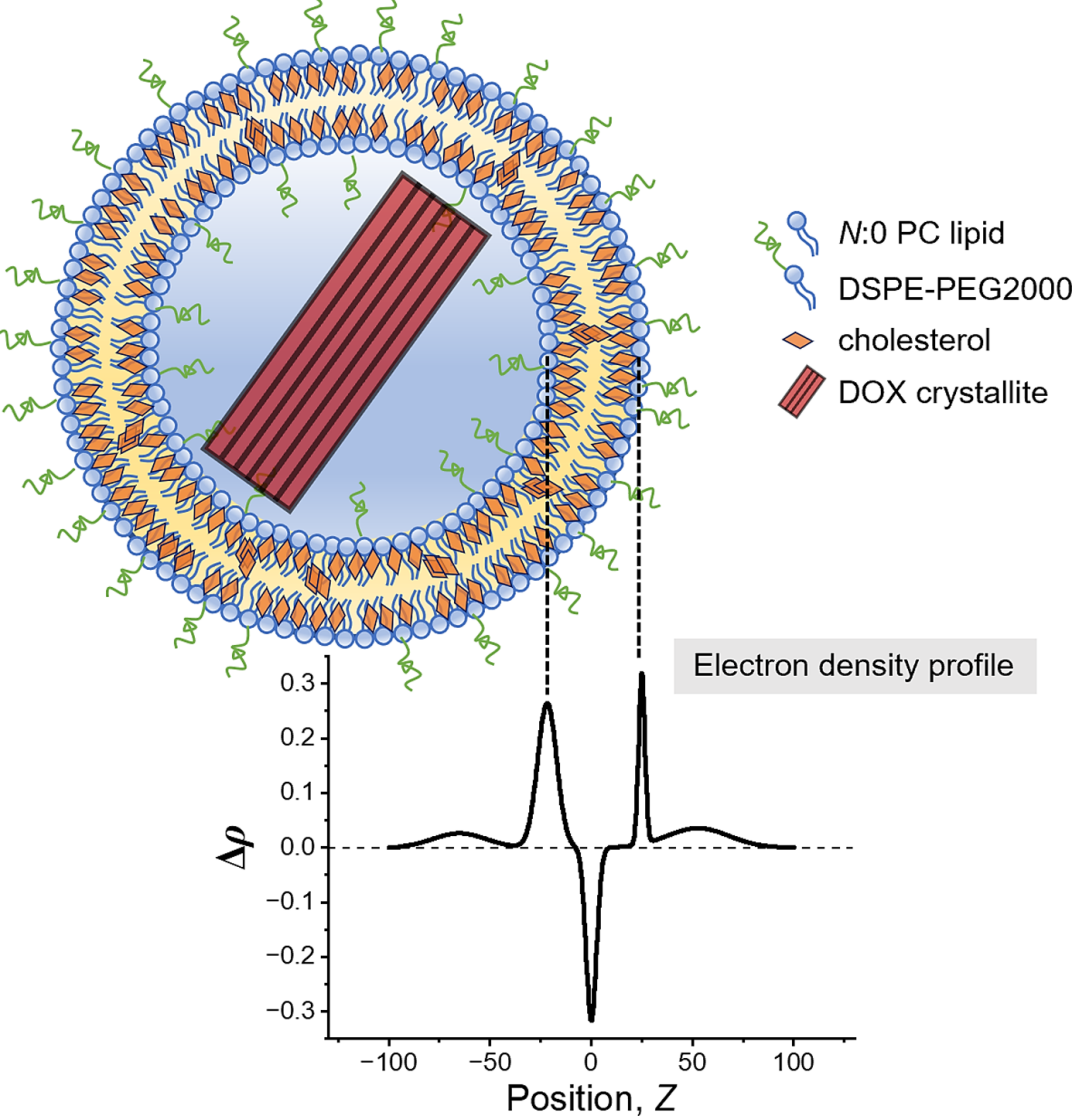
Schematic illustration of the cholesterol-incorporated PEGylated liposome of *N*:0 PC lipids, with a DOX crystallite enclosed in the core region. Below is the contrast electron density profile Δρ across the bilayer, described by a five-layered model in the SAXS data analysis.

For the pristine liposomes, the SAXS data *I*(*q*) were analyzed by
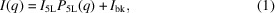
where *I*_5L_ and *P*_5L_(*q*) are the intensity scaling factor and the form factor for the five-layered model, respectively, and *I*_bk_ is the fitted constant scattering background. The five-layered model comprises five Gaussian functions to approximate the gradual transition of the electron density (ED) profile between the headgroup and lipid chain regions of the liposome bilayer as illustrated in Fig. 1 ▸ (Konarev *et al.*, 2021 ▸). Each layer is defined by a Gaussian distribution with a peak position *Z*, standard deviation σ and peak height Δρ for the contrast relative to the electron density of the solvent. The *X+* software (Ben-Nun *et al.*, 2010 ▸) was used for the SAXS model fitting of the pristine liposomes. For comparison, the same SAXS data were also fitted with *P*_5L_(*q*) replaced by the core–multishell model in *SasView 4.2.2* (https://www.sasview.org/; Kline, 2006 ▸), which consists of a spherical core with five concentric shells, each of a uniform electron density, leading to sharp transitions to neighboring shells (Hsu *et al.*, 2023 ▸). The measured liposome sizes obtained from cryo-TEM and DLS were used in the model fitting.

For the DOX-loaded liposomes, the SAXS data were analyzed by
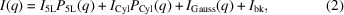
where *I*_Cyl_ and *P*_Cyl_(*q*) are the intensity scaling factor and the cylinder form factor respectively. *I*_Gauss_, a Gaussian peak profile, is included in the fitting model to account for the scattering contribution from the 100 diffraction peak at *q* ≃ 0.22 Å^−1^ from the sulfate-salt DOX crystallites inside the liposome core (Schilt *et al.*, 2016 ▸; Wibroe *et al.*, 2016 ▸). *SasView 4.2.2* was used as the model fitting platform, with the created five-layered model (or the core–multishell model) as a plugin model in the software. Data fitting for the DOX-loaded liposomes was preliminarily performed by applying the structural parameters in *P*_5L_(*q*) obtained from the corresponding pristine liposome sample data analysis, with *I*_5L_, *I*_Cyl_*P*_Cyl_(*q*) and *I*_Gauss_(*q*) being variables. Then, the parameters in *P*_5L_(*q*) were unfixed, and the model fit was performed again with all parameters to obtain the final fitting results. Comparisons of the fitting curves with preliminary and final fitting are detailed in Fig. S1 in the supporting information.

## 3. Results and discussion

### 3.1. Spherical and faceted shapes observed

**Figure 2:**
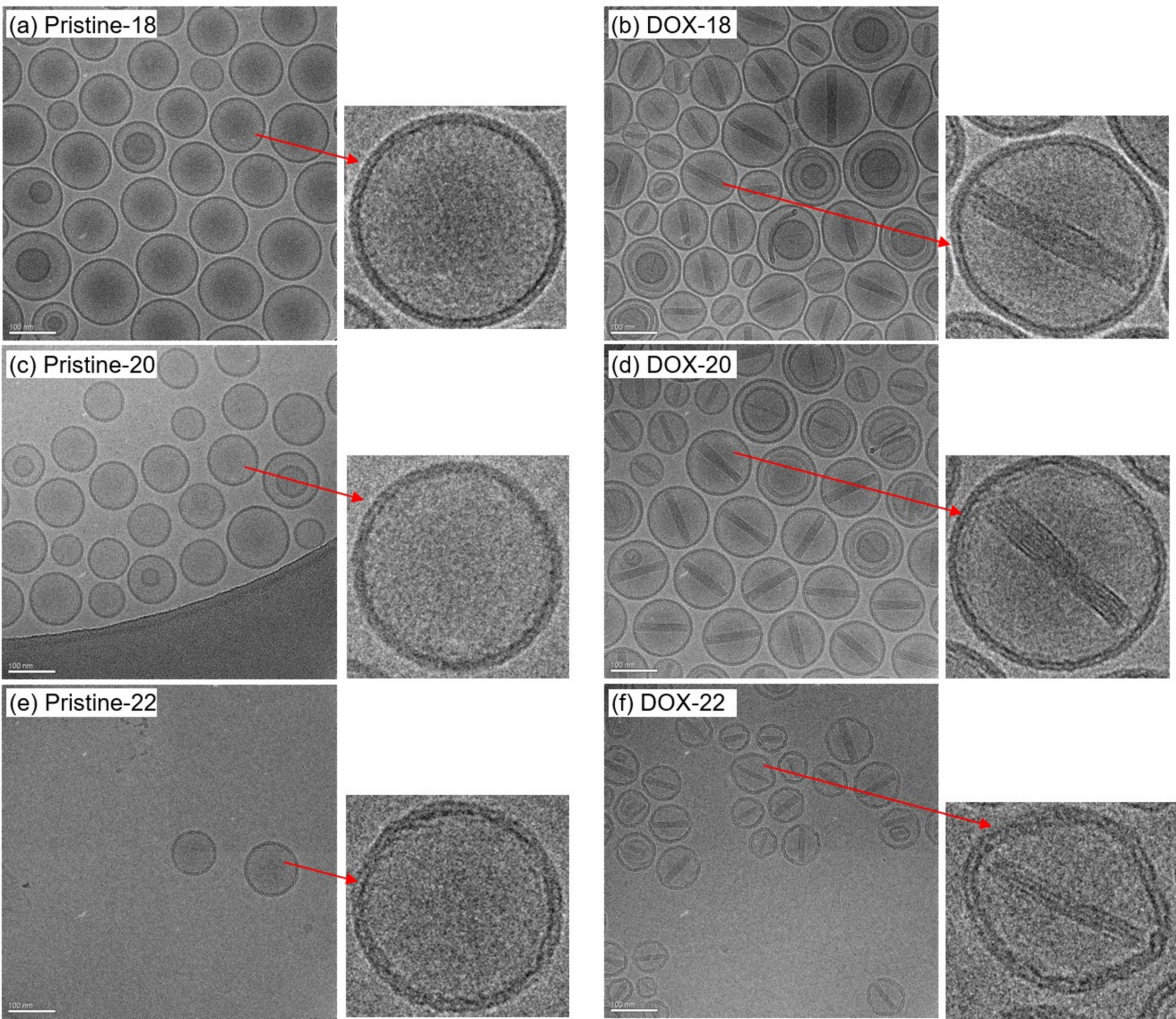
Cryo-EM images of the PEGylated liposomes as pristine and after DOX loading: (*a*, *b*) 18:0 PC, (*c*, *d*) 20:0 PC and (*e*, *f*) 22:0 PC. A scale bar of 100 nm is marked at the bottom-left side. The red arrows indicate the zoom-in views of one selected liposome particle.

The pristine liposomes (Pristine-18, Pristine-20 and Pristine-22) and DOX-loaded liposomes (DOX-18, DOX-20 and DOX-22) were successfully prepared using long-chain saturated phospho­choline lipids (18:0 PC, 20:0 PC and 22:0 PC). To investigate the morphology of the liposomes, cryo-EM was employed, and the resulting images are presented in Fig. 2 ▸. As revealed in the cryo-EM images, the pristine liposomes [Figs. 2 ▸(*a*), 2 ▸(*c*) and 2 ▸(*e*)] predominantly exhibit spherical unilamellar vesicles. Notably, Pristine-22 liposomes demonstrated relatively rough membrane surfaces with locally faceted features, in contrast to Pristine-18 and Pristine-20.

For the DOX-loaded liposomes [Figs. 2 ▸(*b*), 2 ▸(*d*) and 2 ▸(*f*)], rod-like DOX crystallites were visible within the liposomal cores, confirming the successful encapsulation of the drug. Both DOX-18 and DOX-20 liposome particles remain spherical [Figs. 2 ▸(*b*) and 2 ▸(*d*)], while DOX-22 exhibited a much more serious deformation to elongated shapes with more obvious faceted features, significantly deviating from the spherical shape of its pristine state. The observation suggests that the encapsulation of DOX influences the bilayer morphology of, particularly, the 22:0 PC liposome with the relatively longest lipid chains.

### 3.2. Chain-length-dependent liposome size distribution

**Figure 3:**
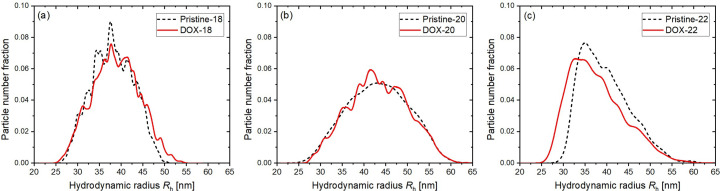
Number density fractions of the liposomes as a function of the hydro­dynamic radius *R*_h_ of the pristine liposome (black dashed curve) and DOX-loaded liposome (red solid curve) of (*a*) 18:0, (*b*) 20:0 and (*c*) 22:0 PC liposomes.

The hydro­dynamic radius *R*_h_, particle mass and particle concentration of the liposomes, without and with DOX loading, were retrieved from the AF4-MALS-DLS-RI measurements (Parot *et al.*, 2020 ▸; Écija-Arenas *et al.*, 2021 ▸). Measurements were performed on the pristine liposomes from the three kinds of PC lipids and their DOX-loaded counterparts. The particle mass for the pristine liposomes can be estimated from AF4-MALS-DLS-RI data, with the concentration converted from the measured RI values with an approximated value of d*n*/d*c* = 0.146 ml g^−1^. Averaged from the full width at half-maximum regions of the particle number fraction profiles shown in Fig. 3 ▸, the number-averaged particle masses are deduced to be 109.4, 158.0 and 102.4 MDa for the liposomes of different lipid chain lengths of *N* = 18, 20 and 22.

The particle size distribution in each type of liposome is presented as the liposome number fraction versus *R*_h_ in Fig. 3 ▸. Notably, all samples exhibit a wide size distribution, expanding from 25 to 60 nm, indicative of a polydisperse nature of the liposomes. The three samples from different *N*:0 PC lipids exhibit different distribution features. Liposomes from 18:0 PC (with a mean *R*_h_ = 37 nm) show a relatively narrow distribution (*ca* 20%), while liposomes from 20:0 PC (with a mean *R*_h_ = 43 nm) show a broad size distribution (*ca* 30%). On the other hand, the *R*_h_ distribution of 22:0 PC liposomes (with the smallest mean *R*_h_ = 35 nm) deviates from the bell shape for a Schulz distribution, having an asymmetric size distribution with a long tail to the high-*R*_h_ side. We note that the liposome size of 20:0 PC is the largest among the three, and liposome 22:0 PC the smallest. Furthermore, comparing the distribution curves of pristine samples and those of the DOX-loaded samples, it is found that the distributions of Pristine-18 and DOX-18 liposomes are about the same; similarity is also found for 20:0 PC liposomes. However, the number-density distribution profile of DOX-22 shows an obvious shift from 35 nm for Pristine-22 to 32.5 nm after DOX loading.

The size and the surface curvature of a liposome are significantly influenced by the lipid composition, which modulates the interactions within the lipid membrane. As the lipids in our study, *i.e.* 18:0, 20:0 and 22:0 PC, are all long-chain saturated lipids, the liposome membranes formed are supposedly less flexible than membranes with shorter-chain or unsaturated lipids. When a membrane becomes more rigid, it may exhibit a reduced ability to bend or form small liposomes with high-curvature-energy surfaces. Therefore, it is generally expected that increasing the aliphatic chain length would lead to larger liposomes, as illustrated by the mean size increase from the 18:0 PC to 20:0 PC liposomes. However, our experimental results show that 22:0 PC liposomes exhibit the smallest size among the three formulations, accompanied by an uneven size distribution. This unexpected result may possibly be associated with intriguing interactions between the high-content cholesterols and the aliphatic chains within the bilayer. As cholesterol is known to increase the fluidity and flexibility of liposomal membranes, in the cases of longer PC chains, membrane rigidity may be better enhanced by promoting ordered packing between the long aliphatic chains and the cholesterol molecules. For the extreme case of 22:0 PC, this ordered packing may lead to the formation of compact, well-ordered structures. As a result of this increase in membrane ordering and condensation, the liposome size may be reduced.

Thus, two conflicting trends seem to influence the liposome size: on one hand, longer aliphatic chains result in greater membrane rigidity and less flexibility, making it harder for the liposomes to form small, highly curved structures; on the other hand, cholesterol complexation with the long aliphatic chains may form phase-segregated domains of different lipid chain packing features (as detailed by WAXS below), leading to membrane condensation and potentially smaller liposome sizes. This interplay between membrane rigidity and condensation likely explains why liposome size does not increase in direct correlation with aliphatic chain length. The uneven size distribution observed for 22:0 PC liposome, with a suppression of smaller liposomes, may be due to the unfavorable high curvatures required for formation of small liposomes with long lipid chains.

### 3.3. Nanostructure of the liposome bilayer

Small-angle and wide-angle X-ray scattering techniques together provide insights into the structural characteristics of the PEGylated liposome bilayer membranes across different length scales. SAXS enables analysis of the bilayer structure, revealing the spatial arrangements of the PC lipids across the unilamellar bilayer, while WAXS probes the molecular-level organization, characterizing the alkyl chain packing and the molecular interactions within the bilayer.

#### 3.3.1. Lipid-chain-length effect

**Figure 4:**
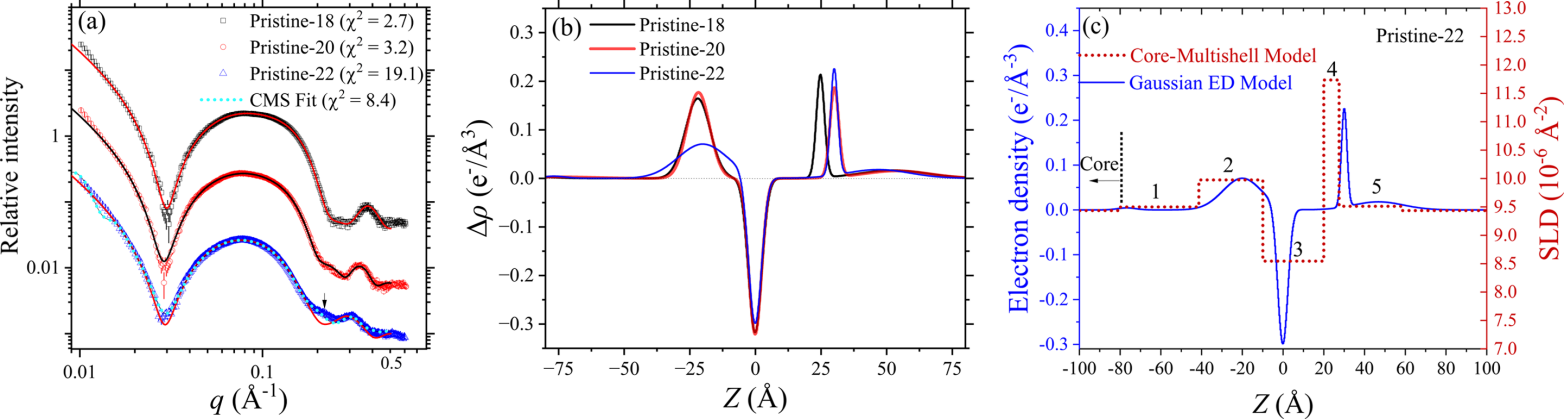
(*a*) SAXS data (from top down) of the three types of Pristine-18, -20 and -22 liposomes, fitted (solid curves with the best-fitted χ^2^ indicated) using the five-layered model. The first two scattering intensity profiles are, respectively, scaled up by a factor of 100 and 10 for clarity in visualization. Note that the not-small χ^2^ values are consequences of fitting the data of small error bars, having a high χ^2^ penalty in more sensitively differentiating parameter values. (*b*) The corresponding best-fitted Gaussian ED profiles, with *Z* = 0 set as the center of the middle layer for the aliphatic chains in the five-layered model and Δρ for the electron density difference with respect to that (0.335 e^−^ Å^3^) of water. Also shown in (*a*) is an improved fitting (dotted curve) using (*c*) the SLD profile of the core–multishell model (CMS) for the Pristine-22 liposomes, with a core radius of 37.7 nm. The five shells are indicated by numbers in (*c*).

For the three pristine liposomes, the SAXS profiles feature the bilayer scattering hump covering the *q* range of 0.02–0.2 Å^−1^ [Fig. 4 ▸(*a*)]; a scattering hump covering the *q* range 0.3–0.4 Å^−1^ is associated with the sublayer of phospho­lipid heads within the bilayer. The data were analyzed by the five-layered model as described in Section 2.5, giving the electron density profile of the bilayer membrane along the radial direction of the liposome (schematically demonstrated in Fig. 1 ▸). Our AF4-MALS-DLS-RI results indicate that all the liposomes studied have diameters larger than 70 nm, validating the employment of the layered model in the sense that the curvature effect of the liposome is negligible in SAXS data analysis (Bouwstra *et al.*, 1993 ▸). Considering the electron density (Table S5) of the components of the liposome, the middle layer with negative electron density contrast Δρ is assigned to the alkyl chains, and the neighboring two layers with positive Δρ are contributed by the sublayer comprising mainly the phospho­lipid headgroups. The additional sublayers extended farther from the sublayer of phospho­lipid heads are associated with the PEGylated DSPE-PEG2000 zone, which is thought to be swollen and to extend into the aqueous solution, thus having a relatively low Δρ value. With the center of the middle layer set to zero, the region with *Z* < 0 presumably represents the inner leaflet of the liposome, and the region with *Z* > 0 represents the outer leaflet. This assignment is consistent with the scattering-length-density (SLD) profiles obtained from independent SAXS data fitting using the core–multishell model (Fig. S5), which consists of a spherical core with five concentric shells as described previously.

Comparing the electron density profiles of Pristine-18 [Fig. 4 ▸(*b*), solid black curve] and Pristine-20 [Fig. 4 ▸(*b*), solid red curve], the most noticeable difference is the marginal shift by 1.0 Å in the maximum position for the outer leaflet, while the rest of the profile exhibits similar electron density distributions. Quantitatively, the peak-to-peak (PtP) distance between the two maxima for Pristine-18 is 47 Å, while PtP = 52 Å for Pristine-20. This increase is consistent with the addition of two –CH_3_ groups on the aliphatic chain, taking the C–C single bond length of 1.54 Å and the bond angle of 109.5°. Namely, the increment corresponds quite well to the estimated length for two times the two additional C–C single bond lengths (∼6 Å). This indicates that the extra methyl groups of the longer 20:0 PC lead to a larger membrane thickness, while the alignment between lipid heads remains intact. On the other hand, the electron density profile of Pristine-22 [Fig. 4 ▸(*b*), solid blue curve] shows a significantly broader peak and a weaker Δρ for the inner leaflet, suggesting a more disordered lipid alignment in the inner leaflet of Pristine-22 compared with Pristine-18 and Pristine-20. The observed disordered alignment could be attributed to the higher membrane curvature of the smaller Pristine-22 particles, combined with the long 22:0 PC aliphatic chains, which likely results in increased steric hindrance in the inner leaflet of the bilayer. This steric hindrance may disrupt the alignment of lipids, leading to the observed disordered structure that is highly asymmetric with respect to the outer leaflet.

Including the low-*q* data in the fitting with the five-layered model of Gaussian density profiles will deteriorate the data fitting in the higher-*q* region (for the structure across the membrane of the liposomes). Therefore, five-layered model fitting is limited to 0.016 Å^−1^ to avoid over-fitting of the low-*q* data, which are increasingly dominated by the large form factor of the spherical liposomes of *ca* 100 nm diameter. As a result, the fitted curves shown in Fig. 4 ▸(*a*) deviate from the low-*q* data. In analyzing the SAXS data of Pristine-22 liposomes, we observed that the five-layered model yields a suboptimal fit, particularly in the region near *q* = 0.2 Å^−1^. To address this, the data were alternatively fitted using a core–multishell model. As shown in Fig. 4 ▸(*a*), the core–multishell model achieves a markedly improved fit, evidenced by a significantly reduced χ^2^ value. Despite this improvement, the resulting SLD and ED profiles from both models remain qualitatively consistent, differing only in minor local structural features [Fig. 4 ▸(*c*)]. The enhanced fit provided by the core–multishell model may indicate the presence of sharper interfaces between the headgroup and aliphatic chain regions—an interpretation that aligns with cryo-TEM observations of 22:0 PC liposomes, which exhibit faceted morphologies suggestive of planarized bilayer domains [Figs. 2 ▸(*e*) and 2 ▸(*f*)].

#### 3.3.2. DOX-loading effect

**Figure 5:**
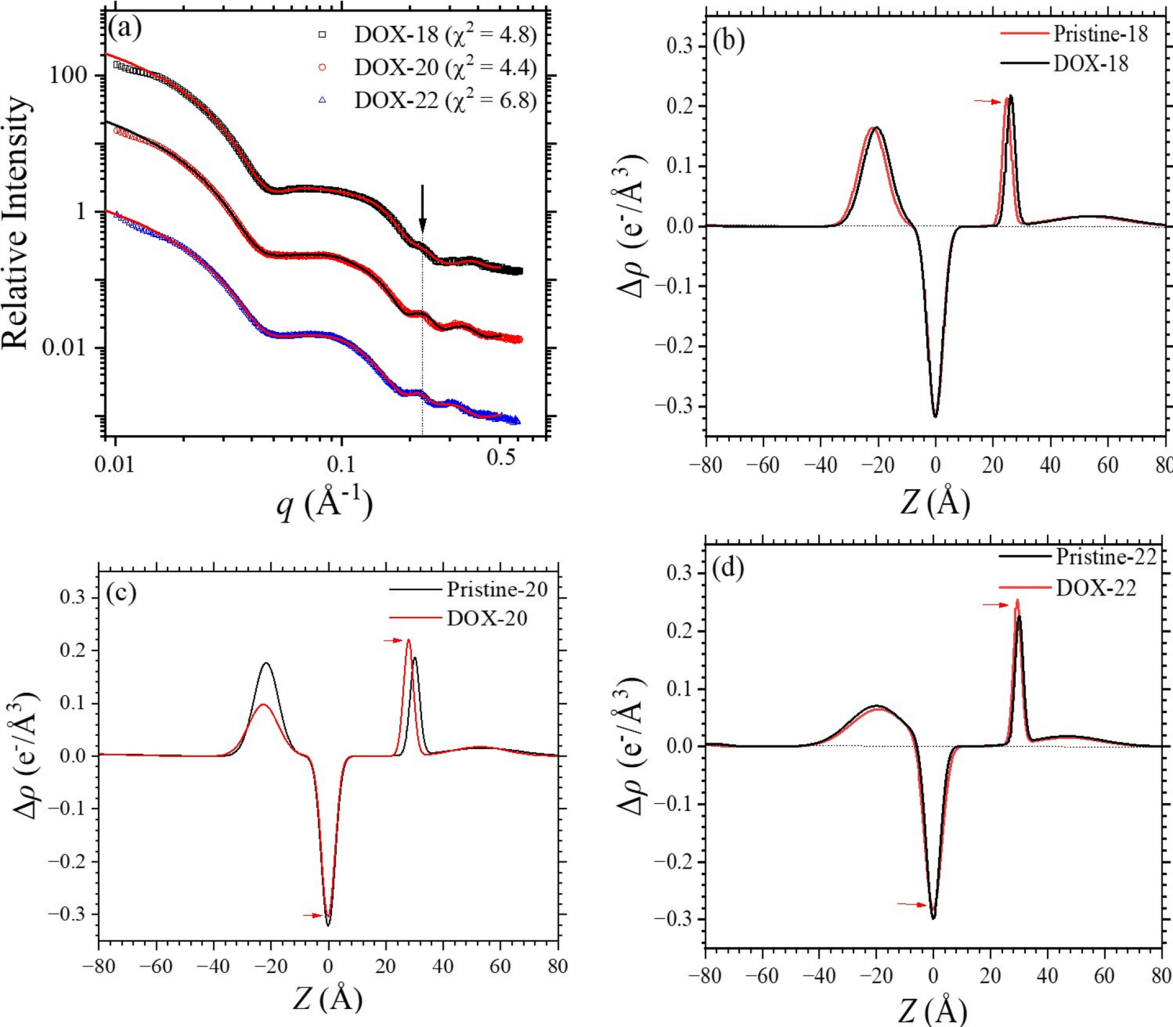
(*a*) SAXS data (top to bottom) of the three types of DOX-18, -20 and -22 liposomes, fitted (solid curves) using the five-layered model. The first two scattering intensity profiles are scaled up for clarity similarly to the pristine cases. The dotted line marks a chain-length effect on the scattering hump position. The corresponding fitted electron density profiles (red curves) of (*b*) DOX-18, (*c*) DOX-20 and (*d*) DOX-22 liposomes. The profiles of the pristine samples (black curves) are also shown for comparison. The arrows selectively indicate changes induced by DOX loading.

For the three types of DOX-loaded liposomes, the SAXS profiles show elevated intensity in the low-*q* region of 0.01–0.03 Å^−1^, compared with those of pristine liposome samples, and a small hump at *q* ≃ 0.22 Å^−1^ [Fig. 5 ▸(*a*)]. These two features are, respectively, contributed by the form factor scattering and crystalline diffraction of the DOX crystallite enclosed inside the liposomes in an acidic ammonium sulfate environment. The corresponding membrane electron density profiles are obtained from the five-layered model fitting, as described in Section 2.5. In the following, the membrane structures of the pristine liposomes are compared with those of the DOX-loaded liposomes for the three kinds of PEG­ylated liposomes.

For 18:0 PC liposome, the electron density profile reveals minimal changes after DOX loading [Fig. 5 ▸(*b*)]. A slight shift of headgroup positions relative to *Z* = 0 is observed, with a marginal increase in Δρ from 0.21 to 0.22 e^−1^ Å^−3^ (relative to water) in the outer leaflet. Critically, the electron density contrast and dispersion width of the optimized five-layered model for the DOX-loaded liposomes remain unchanged, indicating that the DOX-loading procedure did not significantly alter the alignment of lipid heads in either the inner or outer leaflets. In contrast, 20:0 PC liposome exhibited more pronounced structural adjustments [Fig. 5 ▸(*c*)]; the outer leaflet showed a notable increase in Δρ from 0.18 to 0.22 e^−1^ Å^−3^, with a consistent distribution width of approximately 4.0 Å, indicating an improved lipid head alignment. This increase in Δρ could also suggest partial attachment of DOX molecules to the headgroup in the outer leaflet, resulting in the elevated electron density with DOX. Meanwhile, the inner leaflet experiences a significant decrease in Δρ from 0.18 to 0.1 e^−1^ Å^−3^, accompanied by an increase in distribution width by 2 Å, which indicates a deteriorated lipid head alignment. Alternatively, this decrease could indicate cholesterol–DOX–lipid complexation or partial segregation that significantly disturbs the lipid headgroup alignment and reduces the peak electron contrast of the inner leaflet. 22:0 PC liposome exhibits similar changes to 20:0 PC, including an increase of the outer leaflet’s Δρ from 0.22 to 0.25 e^−1^ Å^−3^ after DOX loading [Fig. 5 ▸(*d*)] and a slightly decreased Δρ from 0.07 to 0.06 e^−1^ Å^−3^ in the inner leaflet.

These findings collectively suggest that DOX loading induces increasingly better lipid alignment in the outer leaflet and decreases the ordering of the inner leaflet in liposomes with increasingly longer aliphatic chains (20:0 and 22:0 PC). In contrast, the lipid bilayer structure of 18:0 PC liposome remains intact, indicating fewer DOX-loading effects with the shorter-chain liposomes. The SAXS data of DOX-18 can be fitted comparatively well by the same parameters as used in the SAXS data fitting of pristine liposomes [Fig. S1(*b*), red solid curve]. This suggests that the membrane structure is not much affected by DOX loading. This, however, is not the case for DOX-20 and DOX-22, as illustrated in Fig. 5 ▸.

**Figure 6:**
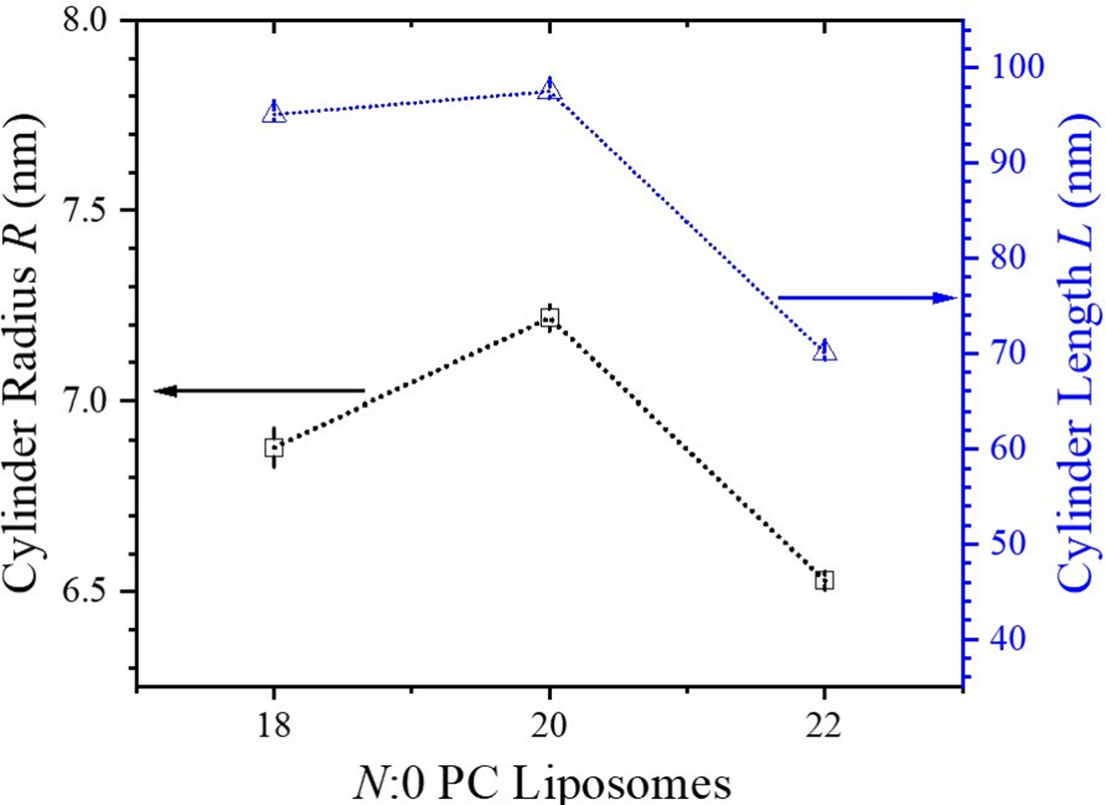
SAXS-determined dimensions of the cylindrical DOX crystallites enclosed inside the three *N*:0 PC liposomes, with *N* = 18, 20 and 22.

The embedded DOX crystallites within the liposomes are of a cylindrical form, as observed in the cryo-EM images [Figs. 2 ▸(*b*), 2 ▸(*d*) and 2 ▸(*f*)]. Accordingly, the SAXS model fitting includes a cylinder form factor to describe the scattering contribution from the DOX crystallites, with converged cross-sectional radii ranging between 6.5 and 7.3 nm and relatively dispersed cylinder lengths spanning from 70 to 100 nm (Fig. 6 ▸). These crystallite shapes and dimensions closely align with the cryo-EM images (Fig. 2 ▸) and are consistent with the previously reported observations in the literature (Schilt *et al.*, 2016 ▸). Notably, the DOX crystallite characteristics corroborate the liposome size trends determined by AF4-MALS-DLS-RI measurements—the largest (20:0 PC) liposomes contain the largest DOX crystallites whereas the smallest (22:0 PC) liposomes enclose the smallest DOX crystallites.

#### 3.3.3. Lipid chain packing

**Figure 7:**
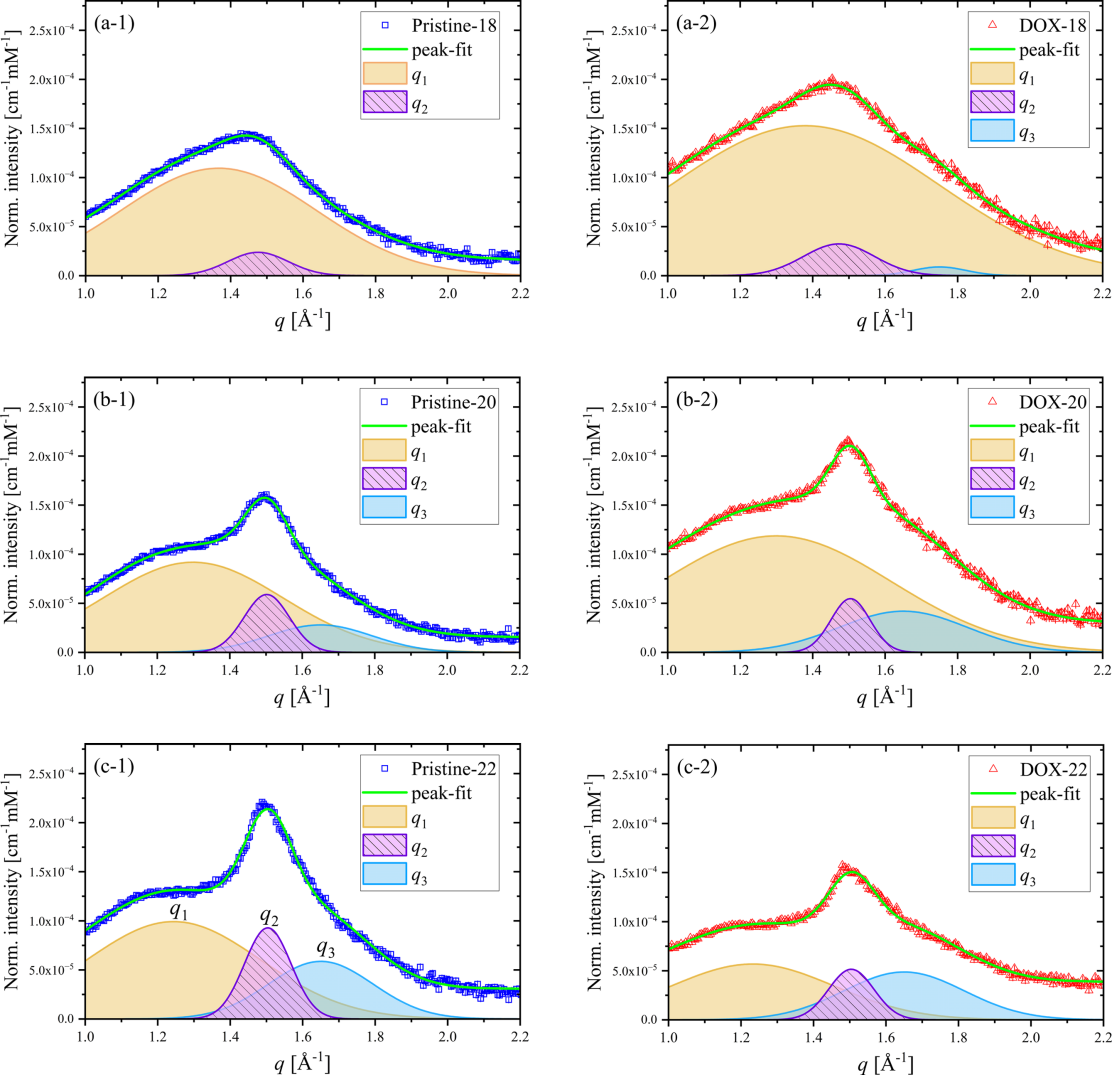
WAXS data of the Pristine-18, -20 and -22 liposomes (left-hand side) and the DOX-18, -20 and -22 liposomes with DOX incorporated (right-hand side). The WAXS data are deconvoluted using the three peaks centered at *q*_1_, *q*_2_ and *q*_3_ as indicated. The concomitantly measured WAXS data are scaled to the SAXS data for absolute intensity and normalized by the liposome concentrations of the sample solutions for quantitative comparison.

WAXS analysis was performed in the *q* range of 1.0–2.2 Å^−1^ to reveal lipid chain packing within the liposomal bilayers. All the WAXS data of the liposomes shown in Fig. 7 ▸ are decomposed into three Gaussian peaks at *q*_1_ ≃ 1.3 Å^−1^, *q*_2_ = 1.5 Å^−1^ and *q*_3_ = 1.65 Å^−1^, or for the Pristine-18 case into two Gaussian peaks. Additional WAXS data of the neat liposomes, without incorporation of cholesterol and DOX, exhibit only the two characteristic *q*_2_ and *q*_3_ peaks (Fig. S6) of the neat lipid chain packing observed in the gel phase of multilamellar lipid bilayers (Tristram-Nagle *et al.*, 1993 ▸). With a high content (50 mol%) of cholesterol incorporated into the liposomes, the *q*_1_ peak emerges (Fig. 7 ▸). Furthermore, the WAXS profiles of the liposomes incorporated only with DOX exhibit similar *q*_2_ and *q*_3_ peaks of the neat lipid chain packing, suggesting that DOX does not interfere directly with the lipid chain packing (Fig. S6). On the basis of these results, the *q*_2_ and *q*_3_ peaks are assigned to a 2D ordered gel phase of the lipid chains, whereas *q*_1_ is attributed to phase-segregated cholesterol-rich domains of relaxed lipid chain packing (Rapaport *et al.*, 2001 ▸). The peak center position *q_i_* (= 2π/*d_i_*) corresponds to a Bragg *d*-spacing with the packing coherent length *L*_c*i*_ deduced from the peak width *w_i_* (= 2π/*L*_c*i*_). All the relevant values deduced are documented in Table 1 ▸.

We further assign the *q*_2_ and *q*_3_ peaks as the 20 and 11 reflections of an orthorhombic packing order (a gel phase) of the lipids, following that reported for multilamellar lipid bilayers (Marsh, 2012 ▸; Sun *et al.*, 1994 ▸; Ruocco & Shipley, 1982 ▸). On the basis of the 20 and 11 reflections of the orthorhombic phase, we deduce the area per lipid *A*_L_ ≃ 44 Å^2^ using *A*_L_ = 2*d*_11_*d*_20_/{cosθ_t_ [1− (*d*_11_/d_20_)^2^/4]} with a chain tilting angle of θ_t_ = 35° from the bilayer normal direction (Tristram-Nagle *et al.*, 1993 ▸). The *A*_L_ value is consistent with those reported for a gel phase (Ruocco & Shipley, 1982 ▸; Tristram-Nagle *et al.*, 1993 ▸). Here, *d*_20_ and *d*_11_ are the Bragg *d*-spacings of the 02-*q*_2_ and 11-*q*_3_ peaks, respectively. As the 11 reflection is weak, the chain packing can also be approximated reasonably well using a distorted 2D hexagonal packing, with the *q*_2_ = 1.50 Å^−1^ peak (*d*-spacing of 4.2 Å) assigned to the 10 reflection (Geisler *et al.*, 2020 ▸; Hsu *et al.*, 2023 ▸). Slightly smaller values of 40–42 Å^2^ are deduced with the *q*_2_ peak alone (Table 1 ▸), using *A*_L_ = 4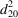/√3 of a distorted hexagonal phase (Geisler *et al.*, 2020 ▸; Hsu *et al.*, 2023 ▸).

Similarly, *A*_L_ values deduced from the *q*_1_ peak position based on a distorted 2D hexagonal packing are about 50–60 Å^2^ for the three types of liposomes (Table 1 ▸). These values are close to that reported for lipids in a fluid phase (Kučerka *et al.*, 2011 ▸; Shih *et al.*, 2018 ▸), suggesting that cholesterol can relax the tight lipid chain packing in the gel phase of the liposome bilayers, forming segregated domains of a fluid phase. As the lipid chain length increased from 18:0 to 20:0, and to 22:0 PC, the WAXS data [Fig. 7 ▸(*a*)] reveal increasingly more prominent *q*_2_ and *q*_3_ peaks for a more enhanced gel phase. Correspondingly, the *q*_1_ peak is systematically suppressed for a reduced fluid phase. Presumably, the enhanced chain–chain self-affinity with the increased chain length in the Pristine-20 and -22 liposomes leads to tighter and more ordered lipid chain packing. The chain-length-dependent decay and growth of the gel and fluid phases are most prominent in the case of Pristine-22. This corresponds to the most broadened inner leaflet and sharpened outer leaflet among the pristine liposomes revealed by SAXS (Fig. 5 ▸). We note that symmetric SLD profiles are often observed for neat liposome bilayers with no cholesterol incorporation (Konarev *et al.*, 2021 ▸; Hsu *et al.*, 2023 ▸). These chain-length-dependent packing features presumably influence the membrane rigidity and membrane permeability on the uptake and release of DOX, as further discussed below.

After incorporation of DOX, the lipid chain packing in the liposomes is influenced to different extents accordingly to the lipid chain length, as revealed from the WAXS data (Fig. 7 ▸ and Table 1 ▸). In general, incorporation of DOX deteriorates the gel phase (decayed *q*_2_ and *q*_3_ peaks) in these liposomes, especially from Pristine-22 to DOX-22. The small broad *q*_3_ hump that emerges in the DOX-18 case might be attributed to an emerging π–π stacking of the aromatic anthracycline rings of the DOX crystallites enclosed inside the liposomes; this is consistent with the DOX crystalline peak at *q* = 0.22 Å^−1^ observed in the SAXS profile [Fig. 5 ▸(*a*)]. Furthermore, DOX-18 and DOX-20 exhibit an enhanced fluid phase (growth in the *q*_1_ peak), respectively, compared with Pristine-18 and Pristine-20, suggesting that DOX may be better incorporated into the fluid phase, especially in the DOX-18 case. Correspondingly, peak-fitting results (Table 1 ▸) disclose that DOX loading leads to a slightly decreased coherent length *L*_c1_ for DOX-18 and DOX-20 liposomes, indicating that DOX intervenes in the cholesterol-rich fluid phase even for less ordered chain packing. In contrast, the *q*_2_ and *q*_3_ peak positions of all the liposomes remain intact upon DOX loading, revealing that the gel phase disfavors incorporation of DOX into the chain packing.

Overall, comparison of the chain packing parameters before and after DOX loading (Table 1 ▸, particularly the integrated peak areas) shows that DOX-22 exhibits the smallest fluid phase (*q*_1_ peak area) and highest gel phase volume (*q*_2_ and *q*_3_ peak areas), revealing the most stiffened bilayer packing structure among the three cases. The longer aliphatic chains with tighter packing form a longer pathway (of a higher barrier) in DOX’s traversal of the bilayers. Meanwhile, the observed significant decrease in WAXS intensity for 22:0 PC liposome suggests that DOX molecules might be trapped in the bilayers, thereby disrupting some of the ordered chain packing domains.

#### 3.3.4. Discussion

The cryo-EM results in Fig. 2 ▸ suggest that the observations in SAXS and WAXS may be correlated. Before the DOX uptake, the cholesterol-rich fluid phase of relaxed chain packing can balance with the gel phase of tighter lipid chain packing in the liposome bilayer for a globally spherical shape of Pristine-22. After DOX loading, the decreased WAXS intensity for DOX-22 (Fig. 7 ▸) indicated a significant loss of ordered chain packing. Owing to the influence of DOX uptake, the long-chain lipids partially revive their general preference in forming neat liposomes of lower-curvature geometries, resulting in the highly asymmetric inner and outer leaflet structures revealed by SAXS (Fig. 5 ▸). Compared with Pristine-20 and -18, the slightly longer lipid chains in DOX-22 contribute to an expanded hydro­phobic region that could play a critical role in forming the faceted liposomes. It is plausible that DOX molecules are trapped inside the additional hydro­phobic domains near the bilayer central zone—particularly in areas not occupied by 50 mol% cholesterol. To stably reside in the hydro­phobic chain zone, DOX may need to form dimers with their hydro­philic moieties facing each other and with the two hydro­phobic anthracycline rings embedded, respectively, into the lipid chain regions of the inner and outer leaflets. The resulting DOX-rich interface zone might decouple direct hydro­phobic chain–chain entanglements/interactions between the inner and outer leaflets of the membrane. Consequently, the two leaflets are more capable of responding independently to their distinct environments: the acidic inside of the liposomes incorporating ammonia sulfate (to diffuse DOX into the inside of the liposome) and the neutral outside of the liposomes containing little ammonia sulfate. Consequently, the outer leaflet of the DOX-22 bilayers may experience fewer conformational constraints than the inner leaflet, enabling it to adopt discrete flat curvatures like freestanding monolayers of highly aligned phosphate headgroups (resulting in the particularly high SLD value revealed). This uncoupling effect could explain the faceted morphology seen in cryo-EM images (Fig. 2 ▸), which suggests a regional loss of the uniform curvature that is typically enforced by interleaflet coupling for bilayer symmetry.

The presence of DOX-rich interfacial zones probably promotes leaflet decoupling, which in turn may hinder further DOX penetration through the liposome bilayers. A sufficient cholesterol content that fully permeates the lipid chains and suppresses the gel phase—such as in the ideal case of DOX-18 with 50 mol% cholesterol—appears to facilitate DOX translocation across the bilayer into the liposome’s interior. This enhancement is attributed to the formation of a cholesterol-rich fluid phase, which provides a more favorable pathway for DOX permeation. In contrast, the DOX-20 liposome represents a transitional case between DOX-18 and DOX-22. It exhibits WAXS characteristics of both formulations and displays the most pronounced structural perturbation in the bilayer upon DOX loading, as evidenced by the SAXS analysis. However, cryo-EM imaging still reveals a generally spherical morphology, without the faceting observed in DOX-22. These findings suggest that the Pristine-20 liposome maintains a delicate balance between the cholesterol-rich fluid regions, the gel-like domains of pure lipid and the intercalated DOX zones. This balance seems sufficient to permit DOX penetration while minimizing excessive decoupling between the inner and outer leaflets, thereby preserving the spherical shape of the liposome.

**Figure 8:**
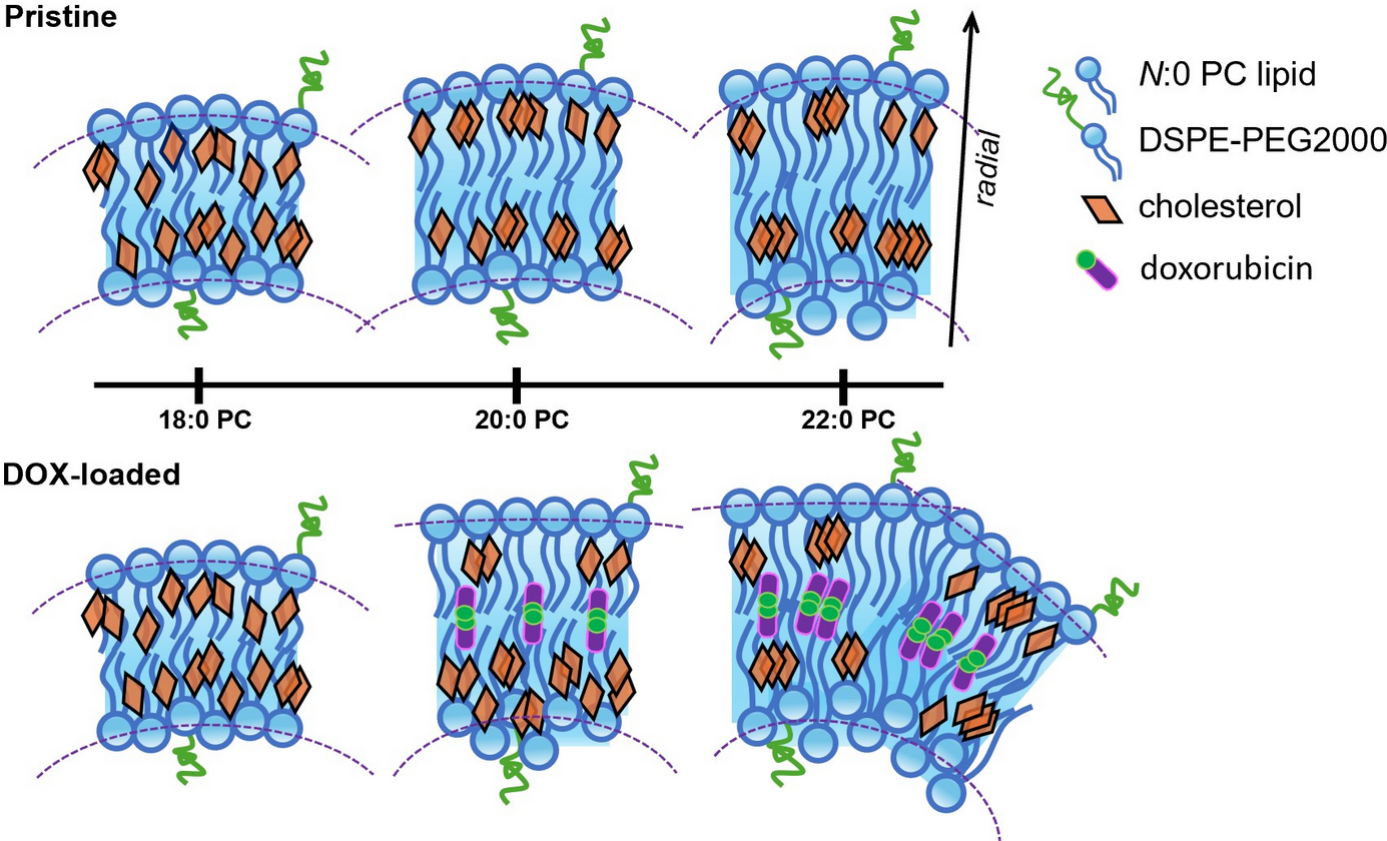
Schematic illustration of the PEGylated liposomal lipid membrane nanostructures and lipid chain packing with 50 mol% cholesterol incorporation based on the SWAXS data analysis. The effects of the aliphatic chain length and DOX intercalation into the liposome bilayers are illustrated through the ordering and disordering of the lipid head and chain regions of the inner and outer leaflets. It is speculated that the DOX molecules become trapped at the center of the hydro­phobic chain region, with their hydro­philic headgroups (represented by green circles) oriented toward one another. The arrow indicates the normal direction of the membranes and the dashed lines outline the curvatures of the leaflets of the membranes.

From the SWAXS data analysis, the membrane structural variations induced by DOX loading in the three types of liposomes can be interpreted through the aliphatic chain-length-dependent DOX incorporation, as schematically illustrated in Fig. 8 ▸. As the lipid chain length increases at a fixed cholesterol content, a progressively larger cholesterol-free hydro­phobic region emerges near the center of the bilayer. This expanded region facilitates the formation of DOX-rich interfacial zones, which in turn promote localized decoupling between the inner and outer bilayer leaflets, resulting in increased structural asymmetry across the membrane.

## 4. Conclusion

Spherical PEGylated liposomes of 18:0, 20:0 and 22:0 PC lipids with chain-length-dependent bilayer structures are observed using cryo-EM, AF4-MALS-DLS-RI, SAXS and WAXS. Experimental results of the pristine liposome are compared with their DOX-loaded counterparts to elucidate the effects from DOX loading on the membrane bilayer structure. As observed via cryo-EM imaging, DOX is loaded successfully into the core region of these three types of liposomes, forming crystallites with a rod-like shape. AF4-MALS-DLS-RI results illustrate a bell-shaped size distribution for 18:0 and 20:0 PC liposomes, while 22:0 PC liposome featured an asymmetric size distribution with its highest population shifts toward the smaller size range. The unexpected result that the 22:0 PC liposomes are the smallest among the three types of liposomes is attributed to high-cholesterol-content effects that alter the lipid chain packing behavior, thus leading to a potential membrane condensation. Upon DOX loading, 18:0 PC liposome exhibits minimal structural modifications, maintaining a largely intact bilayer structure. In contrast, 20:0 PC liposome shows an enhanced electron density profile of the headgroup region of the outer leaflet and a broadened peak for the inner leaflet, suggesting significant DOX participation in the bilayer structure. The 22:0 PC liposome displays relatively small changes in the across-membrane structures upon DOX loading but similar changes in chain packing to 20:0 PC.

Our findings offer ångström-scale insights into the organization of lipid chains within and across liposome bilayers, highlighting the pivotal role of aliphatic chain length in modulating membrane structure and properties. This modulation stems from the intricate interplay between lipid chains, cholesterol and DOX molecules within the bilayer. Notably, liposomes that achieve an optimal balance between aliphatic chain length and cholesterol content—promoting a fluid-phase lipid environment—demonstrate improved membrane permeability and facilitate more efficient DOX incorporation. This effect is most pronounced in 18:0 PC liposomes with 50 mol% cholesterol, which exhibit the highest structural stability upon DOX loading. From a practical standpoint, the presence of limited DOX-rich interfacial zones in 20:0 PC liposomes may provide functional advantages by acting as a buffer for DOX release from the interior to the exterior of the liposome, particularly under environmental conditions opposite to those favoring DOX uptake. Among the three types, DOX loading is found to particularly affect the stability of the 22:0 PC liposome’s cholesterol–lipid bilayers (*i.e.* the structure with the longest aliphatic chains), leading to locally enhanced membrane rigidity for a faceted liposome morphology.

## Supplementary Material

Supporting information file. DOI: 10.1107/S1600576725003577/vg5149sup1.pdf

## Figures and Tables

**Table 1:** Peak-fitting results with the corresponding WAXS data (Fig. 7) of the *N*:0 PC liposomes (*N* = 18, 20 and 22), without (Pristine-*N*) and with DOX loaded (DOX-*N*) *q*_1_ and *q*_2_/*q*_3_ are, respectively, associated with the fluid phase and gel phase of the lipid chain packing. The Bragg *d*-spacing *d_i_* = 2π/*q_i_* with *L*_c*i*_ = 2π/*w_i_*, where *w*_*i*_ is the full width at half-maximum of the *q_i_* Gaussian peak, with the integrated peak area *Q* (in unit of 10^−4^). The area per lipid *A*_L_ is deduced from the *q*_1_ or *q*_2_ peak position, assuming a distorted 2D hexagonal packing.

Sample	*q*_1_ (Å^−1^)/*d*_1_ (Å)/*L*_c1_ (Å)/*Q*_1_/*A*_L_ (Å^2^)	*q*_2_ (Å^−1^)/*d*_2_ (Å)/*L*_c2_ (Å)/*Q*_2_/*A*_L_ (Å^2^)	*q*_3_ (Å^−1^)/*d*_3_ (Å)/*L*_c3_ (Å)/*Q*_3_
Pristine-18	1.37/4.60/10/4.4/49	1.48/4.26/32/0.29/42	Not observable
DOX-18	1.38/4.56/7/7.0/48	1.47/4.27/27/0.39/42	Weak peak
Pristine-20	1.30/4.84/11/3.6/54	1.50/4.18/43/0.59/40	1.65/3.81/19/0.63
DOX-20	1.30/4.84/8/4.7/54	1.50/4.18/50/0.36/40	1.65/3.81/15/0.91
Pristine-22	1.24/5.05/11/3.0/59	1.50/4.18/42/0.75/40	1.65/3.81/19/1.06
DOX-22	1.23/5.10/12/1.5/60	1.50/4.18/43/0.38/40	1.65/3.81/16/0.95
