# Supplementary material for: Changes of the lipid membrane structures caused by chain-length-dependent doxorubicin embedment in PEGylated liposomes
Source: J Appl Crystallogr. 2025 May 29;58(Pt 3):897–908. doi: 10.1107/S1600576725003577 (PMC12135973; doi:10.1107/S1600576725003577)
Supplement: Supplementary file 1 [file j-58-00897-sup1.pdf]

JOURNAL OF
APPLIED
CRYSTALLOGRAPHY

Volume 58 (2025)

Supporting information for article:

Changes of the lipid membrane structures by the chain-length-dependent doxorubicin embedment in the PEGylated liposomes

Jia-Jhen Kang, Zhih-Chen Huang, Li-Wen Tang, Chun-Jen Su, Hua-De Gao, Hsien-Ming Lee and U-Ser Jeng

1. AF4-MALS-DLS-RI measurements

Table S1. The flow parameters for the AF4-MALS-DLS-RI measurements, with the constant cross-flow rate F_c [ml/min] and constant direct-flow rate F_{det} [ml/min]

Elution	Duration [min]	F_c [ml/min]	F_{det} [ml/min]
Elution	1	1	0.4
Focus	2	1	
Focus inject	5	1	
Elution	60	0.4	
Elution inject	3	0	
Elution	10	0	

2. Demonstration of the parameter sensitivities of the 5-layered model with Gaussian electron density (ED) profiles

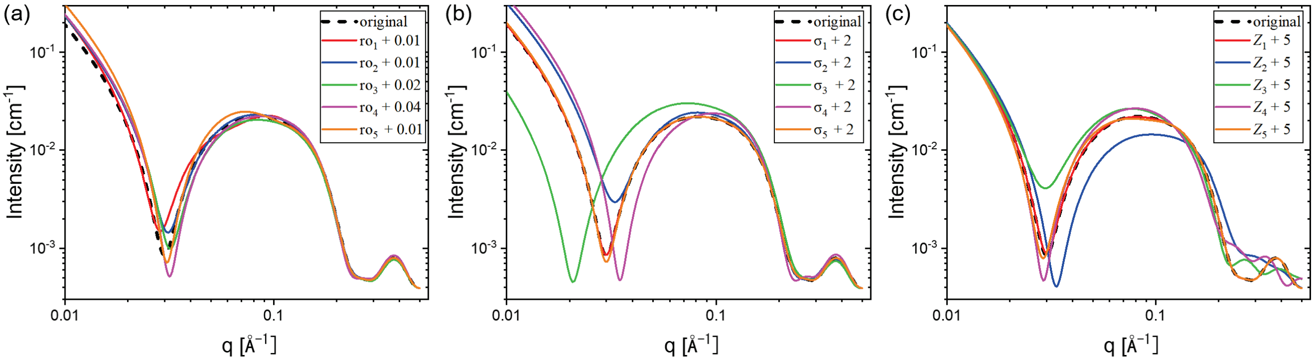

Figure S1 Calculated SAXS curves using the 5-layered model with varying values of a selected fitting parameter (with respect to the best-fitted values, as indicated), while keeping others (see Table S2) the same values as that used in the best-fitting (thick dashed line) for the SAXS data of Pristine-18, including changes in (a) the relative electron density (ED) peak height ro_i of the ith layer 1-5 (in units of $e^{-1}/Å^3$), (b) standard errors of the Gaussian ED distribution σ_i (in units of Å), and (c) center position Z_i of each of the 5 layers (in units of Å). All parameters are listed in Table S2.

Table S2. SAXS best-fitted parameters of the 5-layered model with Gaussian ED profiles

	Parameter	Pristine-18	Pristine-20	Pristine-22	DOX-18	DOX-20	DOX-22
5-layered model , $P_{5L}(q)$ of the liposomes	σ_1	17.3 ± 0.3	13.2 ± 0.2	10.6 ± 1.7	13.9 ± 6.4	35.0 ± 4.1	14.0 ± 1.9
	$\Delta\rho_1$ [Å^{-3}]	0.0029 ± 0.0002	0.0035 ± 0.0001	0.0055 ± 0.0017	0.0031 ± 0.0019	0.0045 ± 0.0012	0.0055 ± 0.0001
	Z_1 [Å]	-85.6 ± 0.2	-81.6 ± 0.1	-76.6 ± 2.1	-71.0 ± 2.3	-87.4 ± 0.4	-85.1 ± 0.1
	σ_2	11.8 ± 0.1	11.2 ± 0.1	22.3 ± 1.3	13.4 ± 0.3	12.1 ± 0.6	22.3 ± 0.1
	$\Delta\rho_2$ [Å^{-3}]	0.1627 ± 0.001	0.1635 ± 0.0010	0.0706 ± 0.0040	0.1073 ± 0.0001	0.0979 ± 0.0013	0.0647 ± 0.0008
	Z_2 [Å]	-21.6 ± 0.1	-22.6 ± 0.2	-19.9 ± 0.7	-21.8 ± 0.1	-22.5 ± 1.9	-19.1 ± 1.9
	σ_3	6.7 ± 0.1	5.3 ± 0.1	5.9 ± 0.2	6.2 ± 0.3	5.5 ± 0.2	7.0 ± 0.35
	$\Delta\rho_3$ [Å^{-3}]	-0.301 ± 0.002	-0.3431 ± 0.0015	-0.3063 ± 0.0048	-0.3038 ± 0.0001	-0.3034 ± 0.0035	-0.2909 ± 0.0043
	Z_3 [Å]	0 ±0.2	0 ± 0.2	0 ± 0.5	0 ± 0.1	0 ± 0.9	0 ± 1.4
	σ_4	3.6 ±0.1	3.8 ± 0.1	3.2 ± 0.1	5.3 ± 0.7	4.1 ± 0.2	3.4 ± 0.1
	$\Delta\rho_4$ [Å^{-3}]	0.2179 ± 0.002	0.1864 ± 0.0018	0.2194 ± 0.0049	0.1875 ± 0.0013	0.2203 ± 0.0075	0.2499 ± 0.0052
	Z_4	24.9 ± 0.1	29.3 ± 0.2	30.2 ± 0.1	24.9 ± 0.1	27.7 ± 1.9	29.5 ± 1.5
	σ_5	33.6 ± 0.1	30.5 ± 0.2	28.8 ± 1.9	10.1 ±1.2	22.1 ± 1.9	27.0 ± 1.1
	$\Delta\rho_5$ [Å^{-3}]	0.0158 ± 0.0001	0.0148 ± 0.0001	0.0180 ± 0.0005	0.0291 ± 0.0068	0.0175 ± 0.0001	0.0156 ±0.0038
	Z_5 [Å]	52.8 ± 0.1	55.9 ± 0.2	47.0 ± 1.3	52.7 ± 0.1	52.8 ± 2.6	47.2 ± 3.0
DOX Cylinder, I_{Cyl}	R [Å]				68.8 ± 0.1	72.2 ± 0.1	66.3 ± 0.3
	$Đ_R$				0.2093 ± 0.0012	0.2277 ± 0.0008	0.218 ± 0.0016
	L [Å]				951 ± 5	975 ± 5	843 ± 5
DOX (100) Crystalline Peak I_{Gauss},	position [Å^{-1}]				0.223 ± 0.001	0.221 ± 0.001	0.217 ± 0.001
	Σ				0.0204 ± 0.0001	0.0248 ± 0.0043	0.0222 ± 0.0052
	χ^2	2.7	3.2	19.0	4.8	4.4	6.8

σ_i: Standard deviation of the Gaussian ED distribution of the ith layer in the 5-layered model.
$\Delta\rho_i$: Electron density contrast of the Gaussian ED distribution of the ith layer in the 5-layered model.
Z_i: Center position of the ith layer in the 5-layered model.
R and L: Cylinder radius and length of the DOX crystals enclosed in the liposomes.
$Đ_R$: Polydispersity of R
Σ: Standard deviation of the Gaussian peak for the DOX crystal (100) reflection.
χ^2: Reduced least squares for goodness of fit

3. Scattering contribution terms of the 5-layered Gaussian ED profiles

SAXS data with the model-fit curves using the 5-layered Gaussian ED profiles, where the scattering contributions in the model are as specified in the text (Eq. 1 and Eq. 2) for pristine liposomes and that with DOX-loaded, respectively.

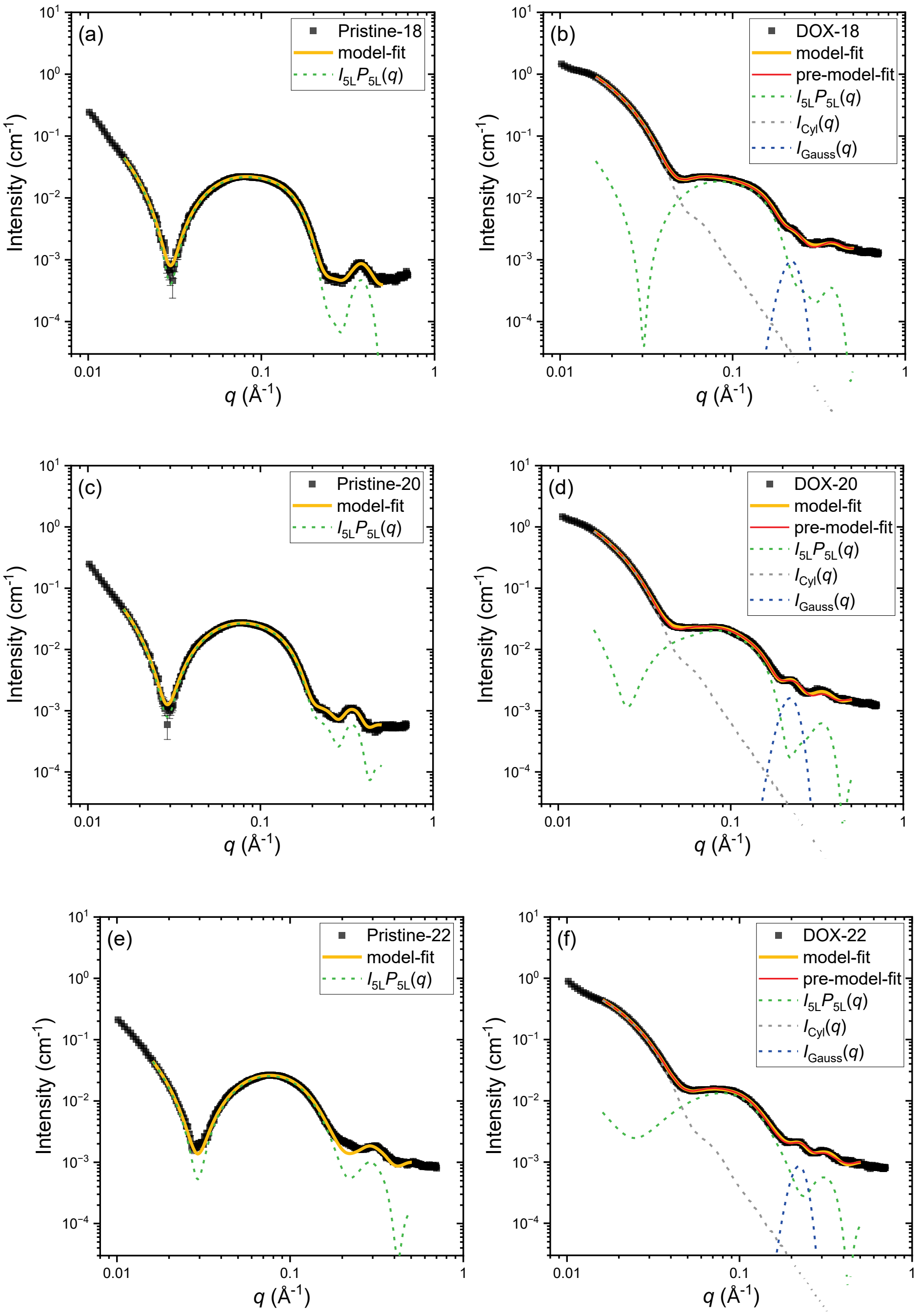

Figure S2 The SAXS data plotted along with the model-fit curves and scattering contribution terms as indicated in the legend. (a) Pristine-18, (b) DOX-18, (c) Pristine-20, (d) DOX-20, (e) Pristine-22 and (f) DOX-22. For the DOX-loaded samples, curves from the pre-model-fit is shown as comparison with the final model-fit. In pre-model-fit, structural parameters in $P_{5L}(q)$ employed the same values obtained from that in the corresponding pristine sample. I_{Cyl}(q) is for the DOX crystal rod scattering, whereas I_{Gauss} is for the (100) reflection of the DOX crystal.

4. Correlation maps of fitting parameters of the 5-layered model with Gaussian ED profiles

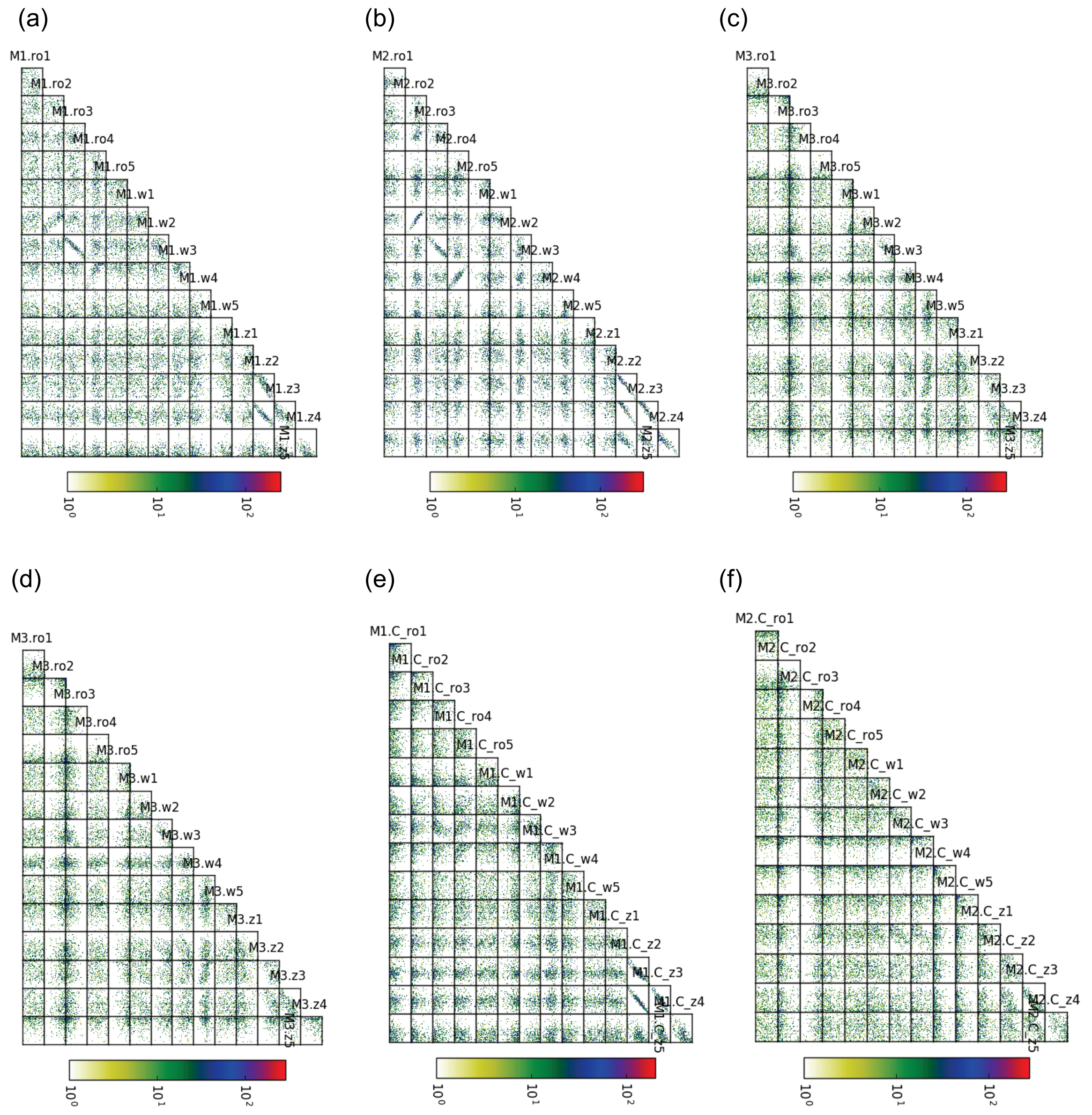

Figure S3 The correlation plot of SAXS fitting parameters for (a) Pristine-18 (b) Pristine-20, (c) Pristine-22, and (d) DOX-18 (e) DOX-20 and (f) DOX-22, where *ro* is the electron density contrast, *w* is the standard deviation of the Gaussian electron distribution and *z* is the centre position of a layer. The correlation maps were generated using the DREAM algorithm in the *Sasview* software. Note that if the parameters are completely uncorrelated then the boxes should contain dots on circles, whereas dots on diagonals indicate strong correlation. Dots forming square blocks indicate that the fit is not sensitive to one of the parameters.

Reference: https://www.sasview.org/docs/user/qtgui/Perspectives/Fitting/optimizer.html#fit-dream

5. Di:22 liposome SAXS data fitting using the core-multishell model with 5 shells

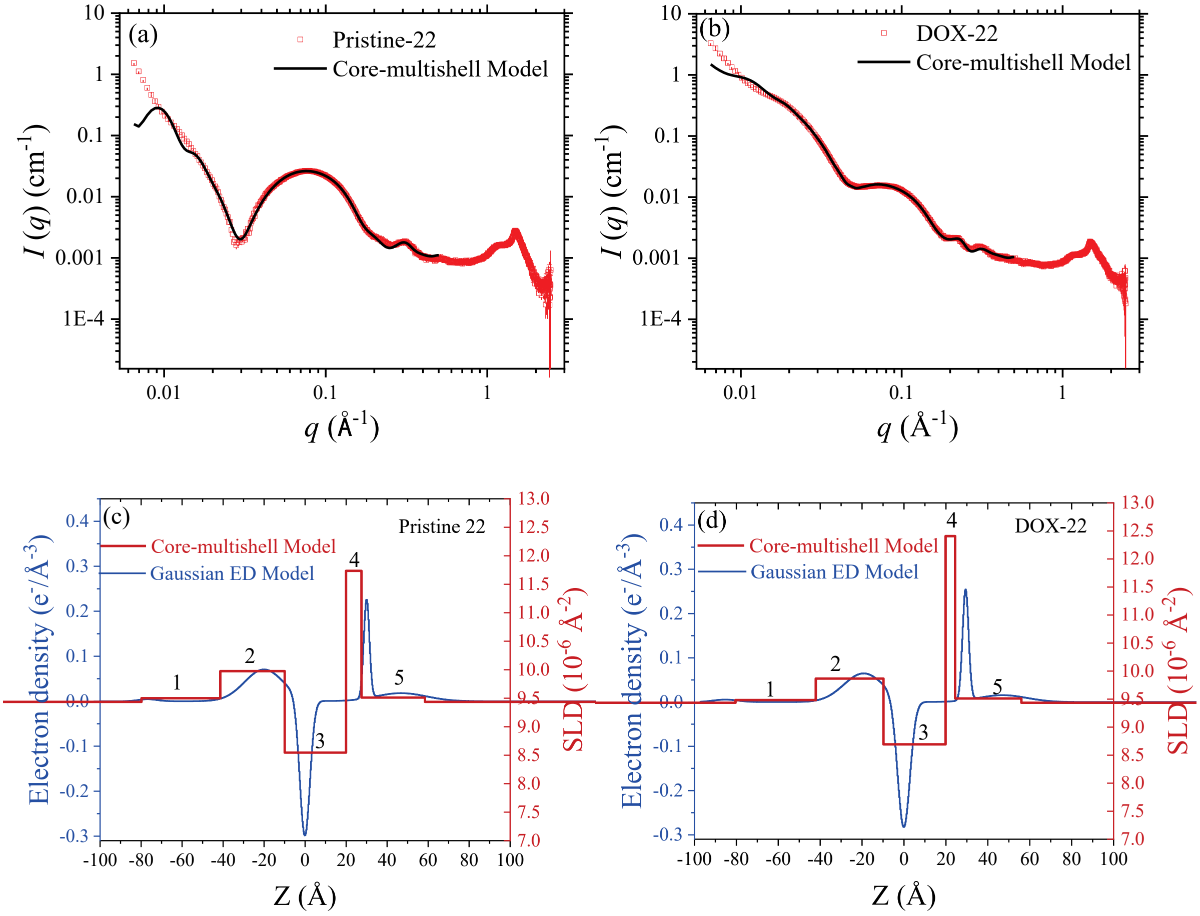

Figure S4 SAXS data of (a) Pristine-22 and (b) DOX-22 liposomes, fitted using the scattering length density (SLD) profile of the core-multishell model with 5 shells as the numbers indicated (solid curves). The scattering contributions from a cylinder form factor and a Gaussian peak for the DOX rod-like crystallite enclosed in the liposome are added to the core-multishell model in the DOX-22 case, following that used in the 5-layered model of Gaussian electron density (ED) profiles. Also shown are (c) and (d) for the best-fitted scattering length density profiles of the liposome structures, compared to the ED profiles best-fitted using the 5-layered model (shown in Figure 4, 5).

Table S3. Best fitted parameters of pristine-18, -20, and -22 liposome (no DOX) using the core-multishell model shown in Figure S4. Note that the core radius is adopted from the peak value measured from the AF4-MALS-DLS-RI results (Figure 3). Note that a q-resolution of 1.0% was used in all fitting to remove the artificial oscillations in the calculated profiles. The χ^2 values were, however, not sensitive to the q-resolution change in 0.1-1.0%.

Pristine-18 $\chi^2 = 3.9$	value	error
Scale	0.0560	0.0002
Background $[cm^{-1}]$	0.00044	3.1478e-6
SLD_core $[10^{-6}Å^{-2}]$	9.435	
Core Radius *Rc* [Å]	371	1
SLD_solvent$[10^{-6}Å^{-2}]$	9.435	
SLD 1 $[10^{-6}Å^{-2}]$	9.470	0.00076
Thickness 1 [Å]	57.0	0.4
SLD 2 $[10^{-6}Å^{-2}]$	10.37	0.004
Thickness 2 [Å]	22.5	0.1
SLD 3 $[10^{-6}Å^{-2}]$	8.191	0.003
Thickness 3 [Å]	23.7	0.1
SLD 4 $[10^{-6}Å^{-2}]$	10.728	0.007
Thickness 4 [Å]	11.2	0.1
SLD 5 $[10^{-6}Å^{-2}]$	9.548	0.00062
Thickness 5 [Å]	41.0	0.1
Rc Polydispersity	0.195	0.07

Pristine-20 χ^2 =3.7	value	error
Scale	0.0695	0.001
Background $[cm^{-1}]$	0.00050	8.7785e-6
SLD_core $[10^{-6}Å^{-2}]$	9.435	
Core Radius *Rc* [Å]	411	3
SLD_solvent$[10^{-6}Å^{-2}]$	9.435	
SLD 1 $[10^{-6}Å^{-2}]$	9.466	0.0043
Thickness 1 [Å]	48.9	1.8
SLD 2 $[10^{-6}Å^{-2}]$	10.162	0.011
Thickness 2 [Å]	26.8	0.3
SLD 3 $[10^{-6}Å^{-2}]$	8.387	0.009
Thickness 3 [Å]	26.7	0.1
SLD 4 $[10^{-6}Å^{-2}]$	11.096	0.035
Thickness 4 [Å]	9.1	0.1
SLD 5 $[10^{-6}Å^{-2}]$	9.524	0.0032
Thickness 5 [Å]	42.5	0.8
Rc Polydispersity	0.172	0.001

Pristine-22 $\chi^2 = 6.5$	value	error
Scale	0.0553	1.0
Background $[cm^{-1}]$	0.00098	1.2567e-5
SLD_core $[10^{-6}Å^{-2}]$	9.435	
Core Radius *Rc* [Å]	377	1
SLD_solvent$[10^{-6}Å^{-2}]$	9.435	
SLD 1 $[10^{-6}Å^{-2}]$	9.499	0.605
Thickness 1 [Å]	38.4	0.5
SLD 2 $[10^{-6}Å^{-2}]$	9.97	5.12
Thickness 2 [Å]	31.4	0.1
SLD 3 $[10^{-6}Å^{-2}]$	8.545	8.438
Thickness 3 [Å]	29.9	0.1
SLD 4 $[10^{-6}Å^{-2}]$	11.736	21.804
Thickness 4 [Å]	7.6	0.3
SLD 5 $[10^{-6}Å^{-2}]$	9.510	0.717
Thickness 5 [Å]	31.0	2.0
Rc Polydispersity	0.176	0.001

Table S4. Best-fitted parameters of the DOX-18, -20, and -22 liposome using the core-multishell model shown in Figure S4. A-series parameters on the left-hand-side table are for the liposome membrane; B- and C-series parameters on the right-hand-side table are for the cylinder form factor and the Gaussian peak of the DOX crystallite enclosed inside the liposome.

DOX-18

Lipsoome, $\chi^2 = 5.4$	Optimized	error
Scale	0.00013	29
Background $[cm^{-1}]$	0.00155	4.11e-6
A_Scale	444.57	9.9979e+7
A_SLD_core $[10^{-6}Å^{-2}]$	9.435	
A_Core Radius *Rc* [Å]	391	5
A_SLD_solvent $[10^{-6}Å^{-2}]$	9.435	
SLD 1 $[10^{-6}Å^{-2}]$	9.491	0.0149
Thickness 1 [Å]	52.5	2.7
SLD 2 $[10^{-6}Å^{-2}]$	10.347	0.0548
Thickness 2 [Å]	21.6	0.5
SLD 3 $[10^{-6}Å^{-2}]$	8.402	0.021
Thickness 3 [Å]	25.5	0.3
SLD 4 $[10^{-6}Å^{-2}]$	11.071	0.098
Thickness 4 [Å]	8.8	0.2
SLD 5 $[10^{-6}Å^{-2}]$	9.56	0.016
Thickness 5 [Å]	39.7	1.4
Core-Rc Polydispersity	0.182	0.007

DOX-20

Lipsoome, χ^2 = 4.4	Optimized	error
Scale	7.289e-5	5.1
Background $[cm^{-1}]$	0.0142	4.3551e-6
A_Scale	1425.7	9.999e+7
A_SLD_core $[10^{-6}Å^{-2}]$	9.435	
A_Core Radius *Rc* [Å]	414	2
A_SLD_solvent $[10^{-6}Å^{-2}]$	9.435	
SLD 1 $[10^{-6}Å^{-2}]$	9.435	0.0001864
Thickness 1 [Å]	44.7	0.1
SLD 2 $[10^{-6}Å^{-2}]$	9.710	0.0040
Thickness 2 [Å]	25.6	0.1
SLD 3 $[10^{-6}Å^{-2}]$	8.762	0.0047
Thickness 3 [Å]	28.4	0.1
SLD 4 $[10^{-6}Å^{-2}]$	11.35	0.0153
Thickness 4 [Å]	7.8	0.1
SLD 5 $[10^{-6}Å^{-2}]$	9.51	0.003
Thickness 5 [Å]	32.3	0.2
Core-*Rc* Polydispersity	0.145	0.001

DOX-22

Lipsoome, $\chi^2 = 9.8$	Optimized	error
Scale	2.41e-5	1.31
Background $[cm^{-1}]$	0.000942	2.77e-6
A_Scale	1836.7	
A_SLD_core $[10^{-6}Å^{-2}]$	9.435	Fixed
A_Core Radius *Rc* [Å]	327	2
A_SLD_solvent $[10^{-6}Å^{-2}]$	9.435	Fixed
SLD 1 $[10^{-6}Å^{-2}]$	9.482	0.183
Thickness 1 [Å]	38.3	Fixed
SLD 2 $[10^{-6}Å^{-2}]$	9.866	1.674
Thickness 2 [Å]	32.3	0.2
SLD 3 $[10^{-6}Å^{-2}]$	8.693	2.89
Thickness 3 [Å]	29.6	0.4
SLD 4 $[10^{-6}Å^{-2}]$	12.4	11.7
Thickness 4 [Å]	4.6	0.9
SLD 5 $[10^{-6}Å^{-2}]$	9.511	0.297
Thickness 5 [Å]	31.6	Fixed
Core-*Rc* Polydispersity	0.08	0.01

DOX-18 DOX Crystallite	Optimized	error
B_scale	1.18	2.66e+5
B_SLD $[10^{-6}Å^{-2}]$	14.393	fixed
B_SLD_solvent	9.435	fixed
B_radius [Å]	66.5	0.6
B_length [Å]	896.0	9.8
C_scale	9.0	2.03e+6
C_peak_pos. [Å]	0.2161	0.0037
C_sigma. [1/Å]	0.0222	0.0021
Rod Radius Polydispersity	0.230	0.001

DOX-20 DOX Crystallite	Optimized	error
B_scale	1.88	1.33e+5
B_SLD $[10^{-6}Å^{-2}]$	14.393	fixed
B_SLD_solvent	9.435	fixed
B_radius [Å]	73.8	0.1
B_length [Å]	700.0	0.2
C_scale	10.3	7.24e+5
C_peak_pos. [Å]	0.2240	0.0006
C_sigma [1/Å]	0.0142	0.0002
Rod Radius Polydispersity	0.230	0.001

DOX–22 DOX Crystallite	Optimized	error
B_scale	3.43	1.87 e+5
B_SLD $[10^{-6}Å^{-2}]$	14.393	Fixed
B_SLD (solvent)	9.435	Fixed
B_Rod Radius [Å]	65	1
B_Rod Length [Å]	900	7
C_scale	28.2	1.53e+6
C_Peak Center *q* [1/Å]	0.2288	0.0005
C_Peak Width [1/Å]	0.0189	0.0005
Rod Radius Polydispersity	0.202	0.001

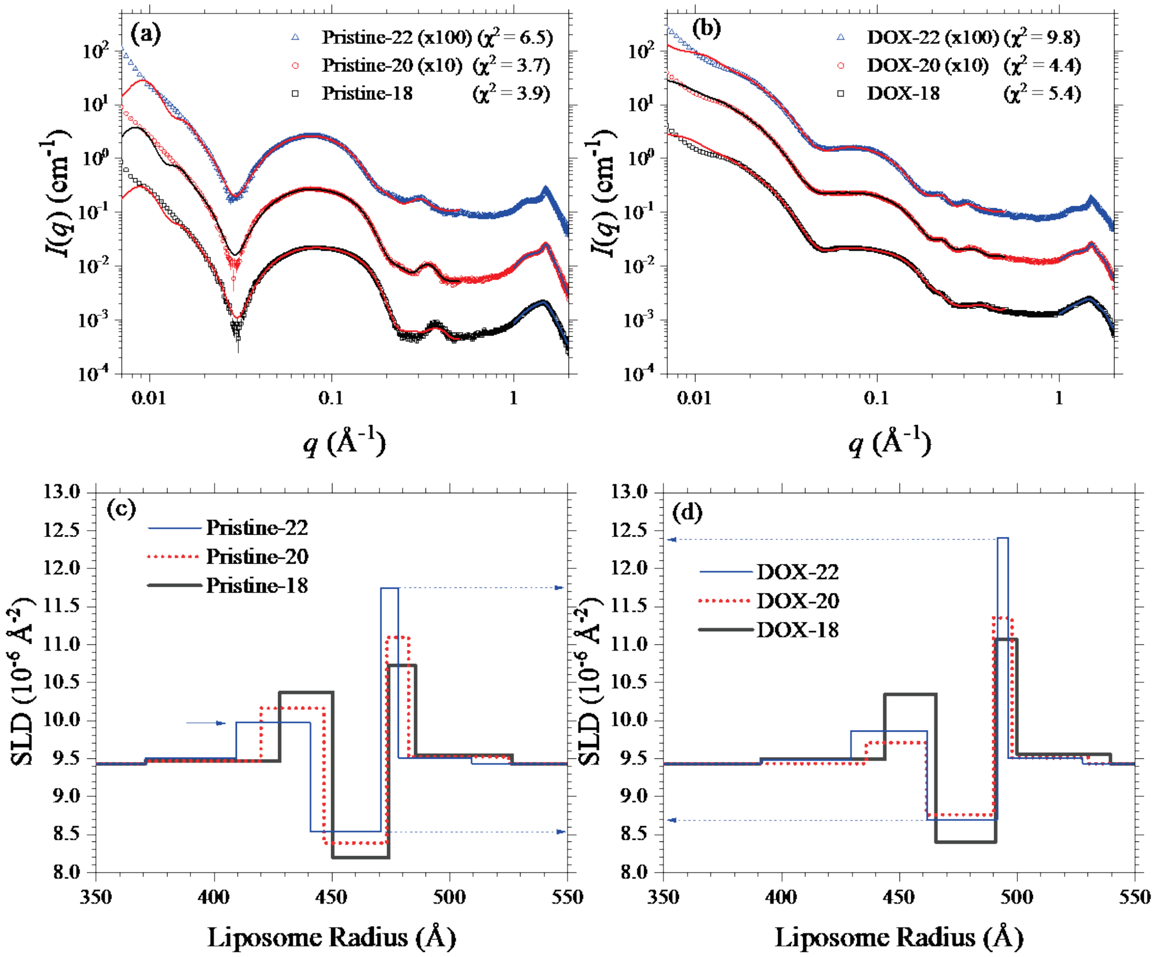

Figure S5 SAXS data of (a) Pristine and (b) DOX-loaded liposomes, fitted using the scattering length density (SLD) profile of the core-multishell model with 5 shells as indicated (solid curves). The scattering contributions from a cylinder form factor and a Gaussian peak for the DOX rod-like crystallite enclosed in the liposome are added to the core-multishell model in the DOX-22 case, following that used in the 5-layered model of Gaussian electron density (ED) profiles. These SLD profiles are qualitatively consistent with the 5-layered model of Gaussian ED profiles; the χ^2 values indicated are from the fitting with the same data q-range (0.016 – 0.5 Å^{-1}) as that done with the 5-layered Gaussian ED model. Also shown are (c) and (d) for the best-fitted scattering length density profiles of the liposome structures, compared to the ED profiles best-fitted using the 5-layered model (shown in Figure 4, 5). Note that the core radius of Pristine–20 and -22 and DOX-20 and -22 are adjusted for better alignments in the three SLD profiles.

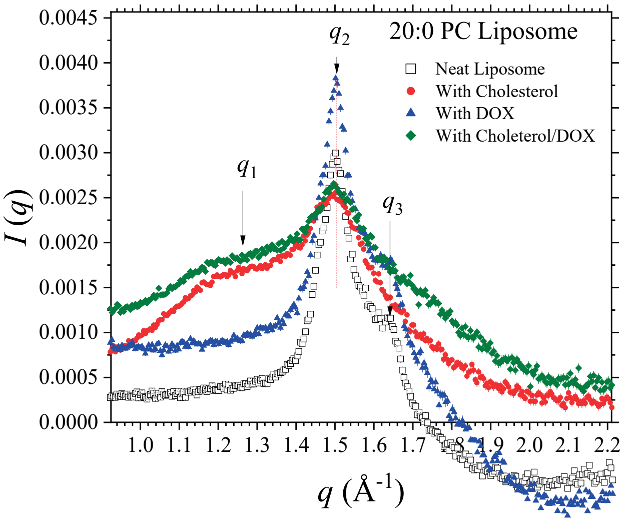

Figure S6 WAXS data of the neat 20:PC liposome, characterized with the $q_2 = 1.50$ Å^{-1} and $q_3 = 1.65$ Å^{-1} peaks. Also shown are the WAXS data for the liposomes with 50% cholesterol added (showing an additional $q_1 \sim 1.3$ Å^{-1} peak), with DOX incorporated, and with cholesterol/DOX incorporated, as indicated.

Table S4. Electron density (ED) and scattering length density (SLD) for the lipid components and cholesterol of the liposomes studied.

	Molar mass [g/moll]	Mass density [g/cm3]	Chemical Formula	ED / SLD [electron/Å^3]/[10^6 Å^{-2}]
Water	18	0.9982 (20 °C)	H_2O	0.333 / 9.39
Cholesterol	387.7	1.05	$C_{27}H_{46}O$	0.352 / 9.92
Phosphate lipid headgroup	183.1	~1.3 - 1.5*	$C_5H_{14}NO_4P$	~ 0.49 / 13.8**
n-octadecane	254.5	0.78	$C_{18}H_{38}$	0.268 / 7.56
n-docosane	310.6	0.78	$C_{22}H_{46}$	0.268 / 7.56
Doxorubicin (DOX)	543.5	1.61	$C_{27}H_{29}NO_{11}$	0.508 / 14.3

*Common for zwitterionic organic compounds like PC

**Higher limit
